# Environmental influences on foraging effort, success and efficiency in female Australian fur seals

**DOI:** 10.1038/s41598-020-73579-y

**Published:** 2020-10-19

**Authors:** Cassie N. Speakman, Andrew J. Hoskins, Mark A. Hindell, Daniel P. Costa, Jason R. Hartog, Alistair J. Hobday, John P. Y. Arnould

**Affiliations:** 1 Deakin University, School of Life and Environmental Sciences, Burwood, VIC Australia; 2 CSIRO Health and Biosecurity, Townsville, QLD Australia; 3 Institute for Marine and Antarctic Studies, University of Tasmania, Hobart, TAS Australia; 4 Ecology and Evolutionary Biology Department, University of California Santa Cruz, Santa Cruz, CA USA; 5 CSIRO Oceans and Atmosphere, Hobart, TAS Australia

**Keywords:** Ecology, Zoology

## Abstract

Understanding the factors which influence foraging behaviour and success in marine mammals is crucial to predicting how their populations may respond to environmental change. The Australian fur seal (*Arctocephalus pusillus doriferus*, AUFS) is a predominantly benthic forager on the shallow continental shelf of Bass Strait, and represents the greatest biomass of marine predators in south-eastern Australia. The south-east Australian region is experiencing rapid oceanic warming, predicted to lead to substantial alterations in prey diversity, distribution and abundance. In the present study, foraging effort and indices of foraging success and efficiency were investigated in 138 adult female AUFS (970 foraging trips) during the winters of 1998–2019. Large scale climate conditions had a strong influence on foraging effort, foraging success and efficiency. Foraging effort and foraging success were also strongly influenced by winter chlorophyll*-a* concentrations and sea-surface height anomalies in Bass Strait. The results suggest increasing foraging effort and decreasing foraging success and efficiency under anticipated environmental conditions, which may have population-level impacts.

## Introduction

Marine predators forage within a highly heterogeneous environment and must respond to changing environmental conditions that influence prey availability at multiple temporal and spatial scales^1^. In order to maximise reproductive success and offspring provisioning, individuals should make foraging decisions that optimise their energy intake (i.e. the quantity and quality of prey) while minimising energetic costs associated with foraging^2^. In colonial breeding central-place foraging species, individuals provisioning young are constrained to foraging within a restricted area surrounding the colony and, thus, are particularly susceptible to shifts in prey abundance and distribution^3,4^. If environmental conditions result in prey patches shifting beyond reasonable energetic limits of the central place, animals need to respond by increasing foraging effort or switching prey type to lower quality prey to account for depleted resources or to abandon offspring in order to access areas of higher productivity^5^. A good example of such impacts is the significant reduction in pup survival and resulting population declines in Galapagos sea lions (*Zalophus wollebaeki*), a species adapted to foraging on unpredictable prey resources^6^, associated with El Niño-induced changes in prey distribution^7^.

Marine ecosystems across the globe have long experienced changes in ocean temperatures, circulation, and nutrient transport^8^. However, anthropogenic activity has caused an accelerated rate of change which is predicted to continue into the future^9^, including shifts in the frequency or severity of large-scale climate events^10–12^. These anticipated climatic changes will alter entire marine ecosystems, with significant compound effect for higher trophic levels including reductions in foraging efficiency and the relocation of foraging zones^13^. For example, changes in sea-surface temperature (SST) can greatly influence juvenile red cod (*Pseudophycis bachus*) survivorship reducing adult recruitment in subsequent years^14^. In turn, this impacts the foraging conditions of the predators that depend on them^14,15^, leading to reduced foraging efficiency, poor reproductive outcomes and/or reduced survivorship^14^. Similarly, reproductive failures in black-legged kittiwakes (*Rissa tridactyla*) and common guillemots (*Uria aalge*) are associated with climate induced changes in phenology of lesser sandeel (*Ammodytes marinus*) in the North Sea^16–18^.

South-eastern Australia has one of the world’s fastest warming ocean regions, largely due to the intensification and southerly extension of the East Australian Current (EAC) and its eddy train^19,20^. The region is projected to undergo further increases in temperature, sea level, salinity and, in some areas, upwelling in the coming decades^21^ and has already experienced oceanographic changes that have altered the diversity, distribution and abundance of species^22^. For example, Thompson et al.^23^ reported a ~ 50% decrease in spring phytoplankton bloom biomass and growth rate in the western Tasman Sea from 1997 to 2007; Johnson et al.^22^ identified shifts in zooplankton communities in the same region between the 1970s and 2000s; and many fish species have extended their southern range limit^24,25^. Such changes are likely to have significant flow-on effects for higher trophic levels (e.g.^26^).

The Australian fur seal (*Arctocephalus pusillus doriferus*; AUFS) population is still recovering from the over-exploitation of the commercial sealing era (1798–1825), with an annual pup production currently estimated at *ca* 28–47% of pre-sealing levels^27,28^. Despite being at less than half of their pre-sealing population, with *ca* 85,500–120,000 individuals^27–30^ and adult female and male body masses of 75 kg and 229 kg, respectively^30,31^, it represents the largest marine predator biomass in south-eastern Australia. Like all otariid seals (fur seals and sea lions), female AUFS adopt a central-place foraging strategy^32^ during the *ca* 10 month lactation period, alternating between 2–11 days foraging at sea and 1–3 days periods on land provisioning their pup^33,34^. Correspondingly, changes in prey diversity, distribution and availability can substantially affect their foraging efficiency, altering offspring provisioning rates and, ultimately, reproductive success^5^.

Australian fur seals are predominantly benthic foragers on the shallow (< 100 m) continental shelf of Bass Strait^33,34^, feeding on a wide variety of prey types comprising bony fish, elasmobranchs and cephalopods^35–37^. Benthic foraging strategies are typically associated with greater effort than pelagic foraging strategies^41,42^. Despite the higher energetic costs associated with benthic foraging, this feeding strategy benefits from more reliable, albeit less productive, prey distributions than is found in pelagic systems^43^. This is important as, despite being influenced by several oceanographic features, including seasonal influences on the movement of waterbodies and upwelling activity, as well as influences of large-scale climate conditions^38–40^, the Bass Strait region is considered nutrient-poor and low in primary productivity^38^. However, several AUFS prey species are pelagic or exhibit pelagic life histories^44–46^ and previous studies have demonstrated temporal variation in the consumption of these species^34,37,43,47^.This temporal variation in consumption is suggestive of changes in the productivity and availability of pelagic prey within Bass Strait between years, with AUFS targeting greater proportions of these prey when they are available.

Considering that air-breathing benthic foragers are subject to elevated physiological demand compared to pelagic foragers^42,43^ and central place foragers are restricted in their ability to adapt to changes in prey distribution and availability, it is likely that AUFS are acutely vulnerable to negative impacts from environmental change. While previous studies have documented relationships between environmental conditions and the diet composition, diving behaviour, body condition, and fecundity in female AUFS^34,48,49^, there is limited information on the environmental factors which influence foraging effort, success and efficiency in AUFS. Such knowledge is crucial for predicting how anticipated changes to their environment may impact the population trajectory of the species and its ecosystem role.

The aims of the present study, therefore, were to examine in female Australian fur seals the influence of local environmental conditions and large-scale oceanographic/climatic indices on: (1) diving behaviour and foraging effort; and (2) foraging success and efficiency. We then used these relationships to discuss how future environmental conditions are likely to influence female AUFS benthic foraging performance.

## Materials and methods

### Animal handling and instrumentation

**Figure 1:**
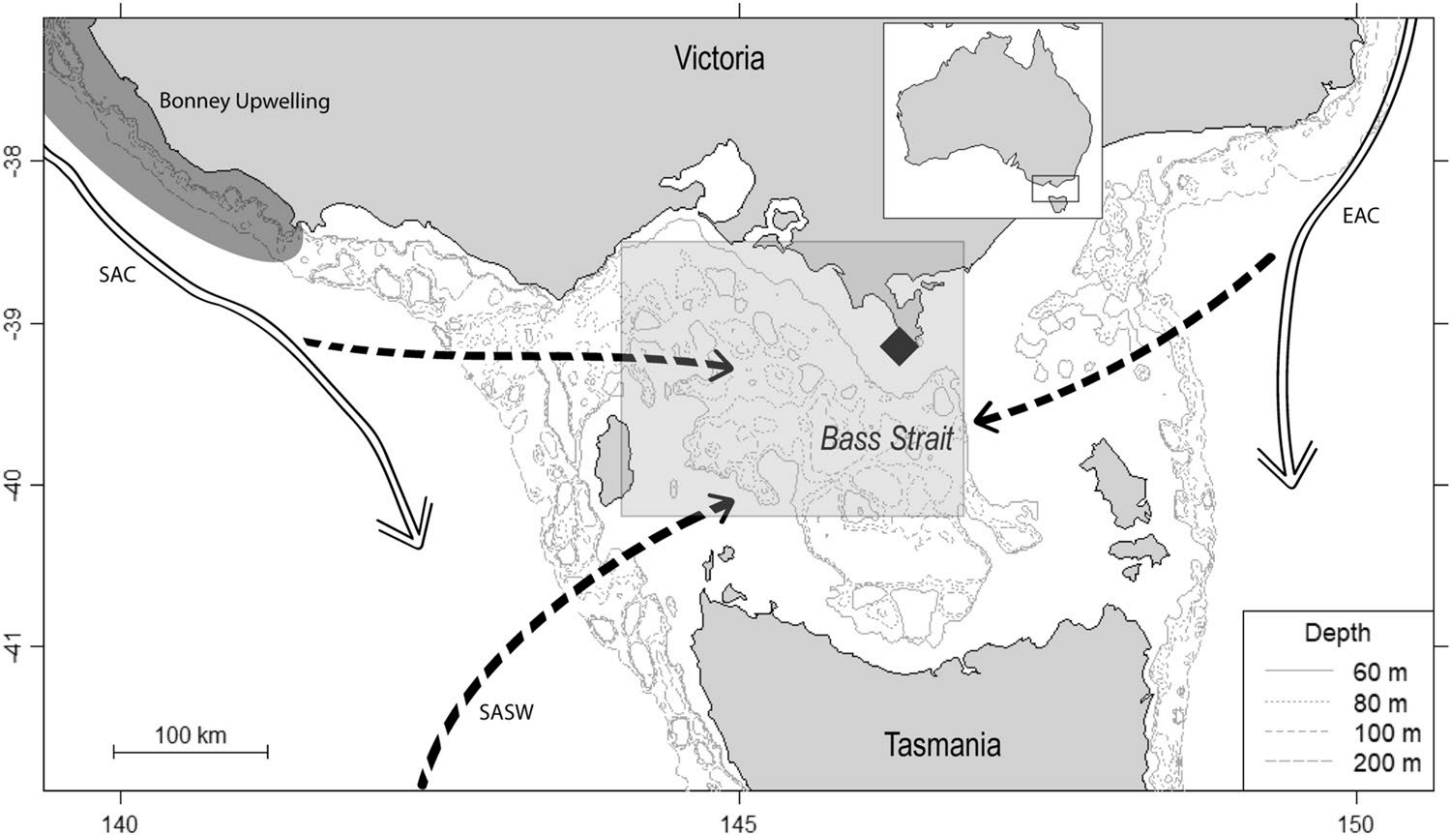
Location of the Kanowna Island breeding colony (♦) within south-eastern Australia and inflow of major water bodies (SAC—South Australian Current; SASW—Sub-Antartic Surface Waters; EAC—East Australian Current) into Bass Strait. Arrows represent current flow and dashed lines represent water flow into Bass Strait. The Bonney Upwelling region is indicated by the shaded grey area and extends into South Australia. Inset map shows the position of the region relative to Australia. The shaded box indicates the region for which local-scale environmental conditions were derived. Map generated using *marmap* (version 1.0.3^110^), *oce* (version 1.1-1^111^) and *ocedata* (version 0.1.5^112^) packages in the R statistical environment (version 3.6.1^51^), and modified using Adobe Illustrator version 23.0.3^113^.

The study was conducted in 1998–2019 at Kanowna Island (39° 10′ S, 146° 18′ E; Fig. 1) in northern Bass Strait, south-eastern Australia. The island hosts a large breeding colony of Australian fur seals with an annual pup production of *ca* 3400^30^ and has been the focus on a long-term research program investigating various aspects of the behaviour, demography and physiology of the species^29,31,33^. Sampling occurred between April and August each year, the period of peak nutritional demand for lactating females^33^.

Lactating females (*n* = 138) nursing pups were selected at random for capture using a modified hoop-net (Fuhrman Diversified, Seabrook, Texas, USA). Prior to 2002, captured individuals were administered an intramuscular injection (*ca* 0.15 mg kg^−1^) of the sedative Midazolam (Hypnovel, Roche Products Pty Ltd., Dee Why, NSW, Australia) before being transferred to a restraint board. In subsequent years, individuals were anaesthetised using isofluorane delivered via a portable gas vaporiser (Stinger, Advanced Anaesthesia Specialists, Gladesville, NSW, Australia) before being removed from the hoop net for processing.

Individuals were then instrumented with a time depth recorder (Mk06, Mk07, Mk08, Mk09, Mk10, or Mk10AF Splash Tag; Wildlife Computers Ltd., Redmond, WA, USA), which can be used to infer diving behaviour, and a VHF transmitter (Sirtrack Ltd., Havelock North, New Zealand) to assist in relocating the animal for recapture. Devices were glued in series along the midline dorsal pelage, just posterior to the scapula, using quick setting 2-part epoxy (Accumix 268, Huntsman Advanced Materials Pty, Deer Park, Vic, Australia & RS Components, Corby, UK). Time depth recorders were programmed to record depth at 1 or 5 s intervals when wet. Individual numbered plastic tags (Super Tags, Dalton, Woolgoolga, Australia) were inserted into the trailing edge of each fore flipper to aid identification. Female AUFS were recaptured as previously described following at least one foraging trip to sea. Devices were removed by cutting the fur beneath the device and individuals were released. Total handling times were < 45 min.

### Data processing

Downloaded data were corrected for drifts in depth readings (zero offset errors) and dive metrics (time of dive, dive duration, post-dive duration, maximum depth, descent and ascent rate, and bottom time) were summarised using the *diveMove* package (version 1.3.5;^50^) within the R statistical environment (version 3.6.1;^51^). A minimum dive threshold of 5 m was used to exclude surface activity and account for differing precisions of the dive behaviour data logger depth sensors across models^49^. As AUFS have been observed to spend several hours in the water for purposes other than foraging (e.g. thermoregulation;^34^), foraging trips were defined as continuous periods of ≥ 6 h in the water in which at least one benthic dive occurred, while haul-out periods were defined as periods of ≥ 10 min out of the water. As GPS locations were not available for all individuals, foraging trips are not necessarily departing and arriving at the breeding colony. Additionally, haul out periods may include periods of ≥ 10 min of relatively motionless surface activity that resulted in salt-water switch drying and, thus, reporting that the individual was on land.

While the majority of AUFS dives are typically classified as benthic, with a distinct descent, bottom and ascent phase, pelagic diving occurs in approximately 15–22% of dives^33,52^. The present study classified benthic and pelagic dives following the methods described in Hoskins et al.^52^. This method derives an index representing the maximum depth achieved for each dive, weighted by proportion of time spent at the bottom of each dive (bottom time). The resulting density distribution of this index is revealed to be bimodal. To classify dives as benthic or pelagic, the distribution is split at the nadir between the two modes, dives falling to the right (i.e. dives that are deep with long bottom times, relative to other dives performed by the individual) are classified as benthic, whereas dives to the left of the nadir are classified as pelagic (Supplementary Table S1). To allow for the influence if individual variation and inter-trip variation (e.g. foraging in areas with different benthic profiles), this classification is performed at the level of individual foraging trip.

For each foraging trip of each individual, the dive duration (min; including the 5 m threshold distance from surface waters), trip duration (h) and benthic dive rate (m h^−1^) was determined as these parameters have been shown to be reliable indicators of foraging effort in AUFS and other otariid seals^43,53,54^. In this study, benthic dive rate was defined as the vertical distance travelled, calculated as:

1 $$b=\frac{\sum_{i = 1}^{N}{d}_{i}+{a}_{i}}{\sum_{i = 1}^{N}{t}_{i}}$$

where *d* represents the descent distance for dive *i, a* represents the ascent distance for dive *i* and *t*_*i*_ represents the sum of each benthic dive duration for the foraging trip.

In addition, while AUFS are considered predominantly benthic foragers, some mid-water foraging does occur^33^. Benthic foraging has been shown to be more energetically costly than pelagic foraging^42^. Therefore, the proportion of dives within a foraging trip that were classified as benthic (PBD) was calculated as an additional index of foraging effort. The PBD also provides a metric for measuring behavioural change, in that increased proportions of pelagic foraging may be indicative of shifting foraging behaviour.

Previously, using animal-borne video equipment, Volpov et al.^55^ confirmed that the diving descent rate (m s^−1^) could accurately predict the probability of prey capture success. As only benthic dives were used in the validation process, foraging success calculations could only be applied to benthic dives in this analysis. Using the parameter estimates for descent rate (4.67) and dive duration (− 6.06) derived from Volpov et al.^55^ for AUFS, we predict the probability *p* that benthic dive *i* is successful as a logistic expression following:

2 $${p}_{i}=\frac{exp\left(4.67\cdot {r}_{i}-6.06\right)}{1+exp\left(4.67\cdot {r}_{i}-6.06\right)}$$

where *r* represents the descent rate (m s^−1^) for dive *i*. From these estimates, a Foraging Trip Success Index (FTSI) for each foraging trip was calculated as the sum of each predicted prey capture success probability *p*_*i*_ divided by the sum of each benthic dive duration *t*_*i*_ for the foraging trip following:

3 $$\mathrm{FTSI} =\frac{\sum_{i = 1}^{N}{p}_{i}}{\sum_{i = 1}^{N}{t}_{i}}$$

where *N* represents the total number of individual dives in the particular foraging trip.

A Foraging Trip Efficiency Index (FTEI) was then calculated as the sum of each benthic dive’s prey capture success probability divided by the benthic dive rate (m h^−1^) as a measure of effort following:

4 $$\mathrm{FTEI}= \frac{{\sum }_{i=1}^{N}{p}_{i}}{b}$$

where *b* represents the total vertical distance travelled (m) while diving during a foraging trip (Eq. 1). Prior to calculation, each of the metrics used in Eqs. (1), (2) and (3) were assessed for correlation, which was found to be low (*r* < 0.3).

### Environmental variables

To investigate environmental influences on foraging effort, success and efficiency in female AUFS, standardised monthly means of climatological variables with known or potential impacts on the prey availability for marine predators within Bass Strait, either directly via changes in productivity or indirectly through impacts on prey recruitment and distribution, were selected for analysis (e.g.^56–58^; Table 1). At the local scale (i.e. within the central Bass Strait region; Fig. 1), mean austral winter (June–August) sea-surface temperature anomaly (SSTa), sea surface chlorophyll-*a* concentration (chl-*a*), zonal (westerly) wind component, and sea-surface height anomaly (SSHa) were obtained as mean monthly values. This area has been shown to be the main foraging area for adult female AUFS from Kanowna Island^33,34,59^.

**Table 1:** Local-scale environmental variables and large-scale climate indices used in the GAMM analyses to investigate influences of environmental fluctuations on Australian fur seal foraging effort, success and efficiency.

Environmental variables	Temporal scale	Abbreviation	Description and main influence	Influence on primary productivity or prey availability	Trends
Indian Ocean Dipole index	Year 1-year lag 2-year lag	IOD IOD_1_ IOD_2_	Major driver of weather in the south-eastern Australian region, associated with changes in sea-surface temperature, zonal wind strength, and pressure systems^64^. During positive IOD events, zonal winds and storm-track activity weaken over southern Australia^64^	Under positive IOD conditions, weakening zonal winds and increasing temperatures may result in decreased productivity in the region^23,96^	The trend towards more positive SAM conditions^92^
Southern Annular Mode	Year 1-year lag 2-year lag	SAM SAM_1_ SAM_2_	Major driver of weather in the region, associated with changes in zonal wind strength and pressure systems^23^. In south-eastern Australia, negative SAM conditions are associated with stronger zonal (westerly) winds and low pressure systems, while positive SAM conditions are associated with warming and weaker zonal winds^23,96^	The weakening of the SAC under positive SAM conditions is associated with reduced flow of nutrient-rich waters into Bass Strait^10,23,96^	The trend towards more positive SAM conditions, which is expected to continue^10^
Southern Oscillation Index	Year 1-year lag 2-year lag	SOI SOI_1_ SOI_2_	The El Nino Southern Oscillation (ENSO) is typically measured by the Southern Oscillation Index (SOI) and is a major driver of weather in the region, associated with changes in sea-surface temperature and primary productivity^85^	Winter El Nino conditions may weaken the Subantarctic Surface Water (SAC) and enhance upwelling in south-eastern Australia in the following summer^85^	Increasing frequency of extreme ENSO events^11^
Chlorophyll *a*	Winter Spring 1-year lag Spring 2-year lag	Chl-*a*_winter_ Chl-*a*_spring1_ Chl-*a*_spring2_	Indicator of primary productivity within a region^87^	Shifts in primary productivity result in shifts in prey availability^87^	Greatly influenced by wind strength and sea-surface temperature, and the large-scale climate conditions that influence these variables^87^
Sea-surface temperature anomaly	Winter Spring 1-year lag Spring 2-year lag	SSTa_winter_ SSTa_spring1_ SSTa_spring2_	Indicator of the influence of different water masses through Bass Strait^39^. Bass Strait is influenced by several water masses: warm South Australian Current (SAC) driven along the south-coast of Australia by westerly winds; cool, nutrient rich Subantarctic Surface Waters (SASW) driven by northward movement of the subtropical convergence in winter; the nutrient poor East Australian Current (EAC) driven by south-easterly winds following southward movement of the subtropical ridge in summer^39^	Warming surface waters stabilise the upper ocean and reduce nutrient supply to the surface, reducing the primary productivity in the region and influencing species distribution^39^	Average sea-surface temperatures in south-eastern Australia are projected to be 2 °C higher by 2050 than the 1990–2000 average^21^
Sea-surface height anomaly	Winter Spring 1-year lag Spring 2-year lag	SSHa_winter_ SSHa_spring1_ SSHa_spring2_	Indicator of eddy energy in a region^80^	Associated with changes in prey abundance, particularly pelagic prey^56^	Sea levels are projected to increase over coming decades^9^
West–east wind component	Winter Spring 1-year lag Spring 2-year lag	Wind-*u*_winter_ Wind-*u*_spring1_ Wind-*u*_spring2_	Primary driver of water flow of nutrient rich waters from the Bonney Upwelling region into Bass Strait^39^	Increased flow of nutrient-rich waters from the Bonney Upwelling region can result in greater prey availability, particularly of pelagic prey, within the Bass Strait region^58^	Zonal wind bands and subtropical ridge have shifted poleward by 5° over the last century and are expected to continue^109^

Sea-surface temperature anomalies were calculated from monthly mean SST derived from CSIRO 3 days composite SST (1996–2008; from https://www.marine.csiro.au/remotesensing) and RAMSSA (2009–2019)^60^. Monthly means of chl-*a* were derived from SeaWiFS (1997–2010)^61^ and MODIS (2011–2019)^62^ NASA satellite based ocean colour imagery. Zonal wind component and SSHa were extracted from NCEP and synTS, respectively. All local-scale environmental variables were extracted at 4–9 km resolution. Marine heatwave duration and intensity^63^ were also considered but were correlated with the other local-scale variables and so were excluded from further analyses.

Large-scale environmental indices, including the Southern Oscillation Index (SOI), Southern Annular Mode (SAM) and the Indian Ocean Dipole mode (IOD) were obtained as monthly anomaly values from the National Oceanic and Atmospheric Administration (https://psl.noaa.gov) and averaged to create annual values. These large-scale indices can influence primary productivity in Bass Strait^64,65^, with potential effects on higher trophic levels (prey) and hence the foraging efficiency and success of marine predators.

The primary spawning time for many AUFS prey species occurs during the austral spring (September–November)^15,66,67^. However, juvenile prey survival can be greatly affected by environmental fluctuations, impacting adult recruitment in subsequent years^12,13^. Hence, to investigate the potential influence of lagged conditions on the foraging behaviour and success of AUFS, 1- and 2-year lagged conditions were included in the analyses. Local-scale variables included 1- and 2-year lagged austral spring, while large-scale variables included 1- and 2-year lagged yearly means (Table 1).

### Statistical analyses

All statistical analyses were conducted in the R statistical environment version 3.6.1^51^. Data exploration was conducted following the protocols described in Zuur, Ieno & Elphick^68^. Prior to analyses, covariates were assessed for collinearity by calculating variance inflation factors (VIF) and correlation coefficients using the *AED* package version 1.0^69^. Based on initial exploratory analyses and the nested nature of the data, trip duration, proportion of time spent diving, FTSI and FTEI were each modelled using Linear Mixed Effects models (LME) using the *nlme* package (version 3.1-140;^70^), with individual fur seal was used as a random intercept. Initial exploratory analyses suggested both linear and non-linear relationships between explanatory variables and dive duration and benthic dive rate. As such, these response variables were modelled using Generalised Additive Mixed Effects Modelling (GAMM). GAMMs were fit with a Gaussian distribution with ‘log’ link function using *mgcv* version 1.8-31^73–73^. The proportion of benthic diving was fit with GAMM using a quasibinomial distribution with a ‘logit’ link function. A random intercept effect of individual fur seal, nested in year, was included in all GAMMs. Smooth terms were fitted to all predictor variables using penalised thin-plate regression splines. The ‘gam.check’ function in *mgcv* was used to determine that the number of knots allowed for enough wiggliness in each smooth term. Autocorrelation of residuals were assessed using ACF plots, which indicated that autocorrelation structures were not needed. To avoid over-parameterisation, models were fit for large- and local-scale environmental indices separately. This method allows both categories to be identified as important, even if correlated. This is important as local-scale conditions can be influenced by large-scale climate conditions, allowing us to try disentangle the influences of environmental parameters.

Candidate models were identified via the ‘*dredge*’ function (*MuMIn* package version 1.43.6^74^). Models selection was achieved by comparing null, maximal and candidate models using Akaike’s Information Criterion corrected for small sample sizes (AICc) and the difference in AICc (ΔAICc) with a threshold difference < 4^75^ to find the optimum model. The optimal model was then refitted with REML to extract model estimates and significance of smooth terms. Unless stated otherwise, data are presented as mean ± SE.

### Ethics statement

All research methods were conducted in accordance with the regulations of the Deakin University Animal Ethics committee (Approval A33/2004, A16/2008, A14/2011, B16/2014, B04/2017), Macquarie University Animal Care and Ethics Committee (Approval 97001, 2000/004), University of Melbourne Animal Experimentation Ethics Committee (Approval 01146) and Department of Sustainability and Environment (Victoria, Australia) wildlife research permits (10000187, 10000706, 10001143, 10001672, 10002269, 10005362, 10007153, 10008286 and 10005848).

## Results

### Diving behaviour and foraging effort

Data were obtained from a total of 138 individuals, with deployment durations ranging 2.7–140.5 days (32.2 ± 2.6 days). Individuals performed an average of 7.0 ± 0.6 foraging trips (Supplementary Table S2). A total of 970 foraging trips were recorded with trips ranging between 6.1 h and 9.9 days (2.7 ± 0.1 days), with significant variation between years (ANOVA: F_20_ = 18.020, *p* < 0.001). Individuals spent an average of 43.8 ± 0.4% of their time at sea diving, completing an average of 244.5 ± 3.5 dives per day, with a total of 601,705 dives recorded.

Individuals had a mean modal dive depth of 59.7 ± 0.9 m. Modal dive depths for benthic dives ranged from 28 to 102 m, representing the range of bottom depths in Bass Strait (Fig. 1). However, three individuals reached maximum dive benthic depths between 119 and 256 m, indicating that they were foraging at the shelf edge. Individuals had an average dive duration of 2.80 ± 0.03 min and an average dive rate of 979.3 ± 10.6 m h^−1^ throughout foraging trips. Significant variation in dive rate was observed among years and between individuals (ANOVA: F_20_ = 8.231, *p* < 0.001 and F_118_ = 4.051, *p* < 0.001, respectively). The proportion of benthic diving also varied significantly among years and individuals (ANOVA: F_118_ = 8.764, *p* < 0.001 and ANOVA: F_20_ = 16.541, *p* < 0.001, respectively), with an average of 78.2 ± 0.7% benthic dives (Supplementary Tables S2, S3).

### Environmental conditions and their influence on foraging effort

Interannual variation was observed in all local-scale environmental conditions and large-scale climate indices assessed (Supplementary Table S4). Mean winter chl-*a* concentrations in the Bass Strait region ranged from 0.48 to 0.85 mg m^−3^ between 1998 and 2019, with concentrations typically higher than average (0.61 ± 0.02 mg m^−3^) since 2011. The mean winter zonal wind component was strongest during two peaks (2002–2005 and 2015–2019), averaging 4.23 ± 0.19 m s^−1^ indicating a tendency toward westerly winds. Mean winter sea-surface temperature anomalies (1.90 ± 0.08 °C) were typically highest in years with positive SAM or SOI (e.g. 1998, 2011 and 2015). Mean yearly IOD (0.25 ± 0.04) was negative during 2 years (1996 and 2005). Three of the six highest IOD events occurred alongside strong negative SOI conditions (1997, 2015 and 2019, the three strongest negative SOI years), while one occurred in conjunction with strong positive SOI conditions (2011). The three strongest positive SOI events occurred within a 4-year period (2008–2011). The SAM conditions during the study period were typically neutral, though had a tendency towards positive (0.50 ± 0.11).

**Figure 2:**
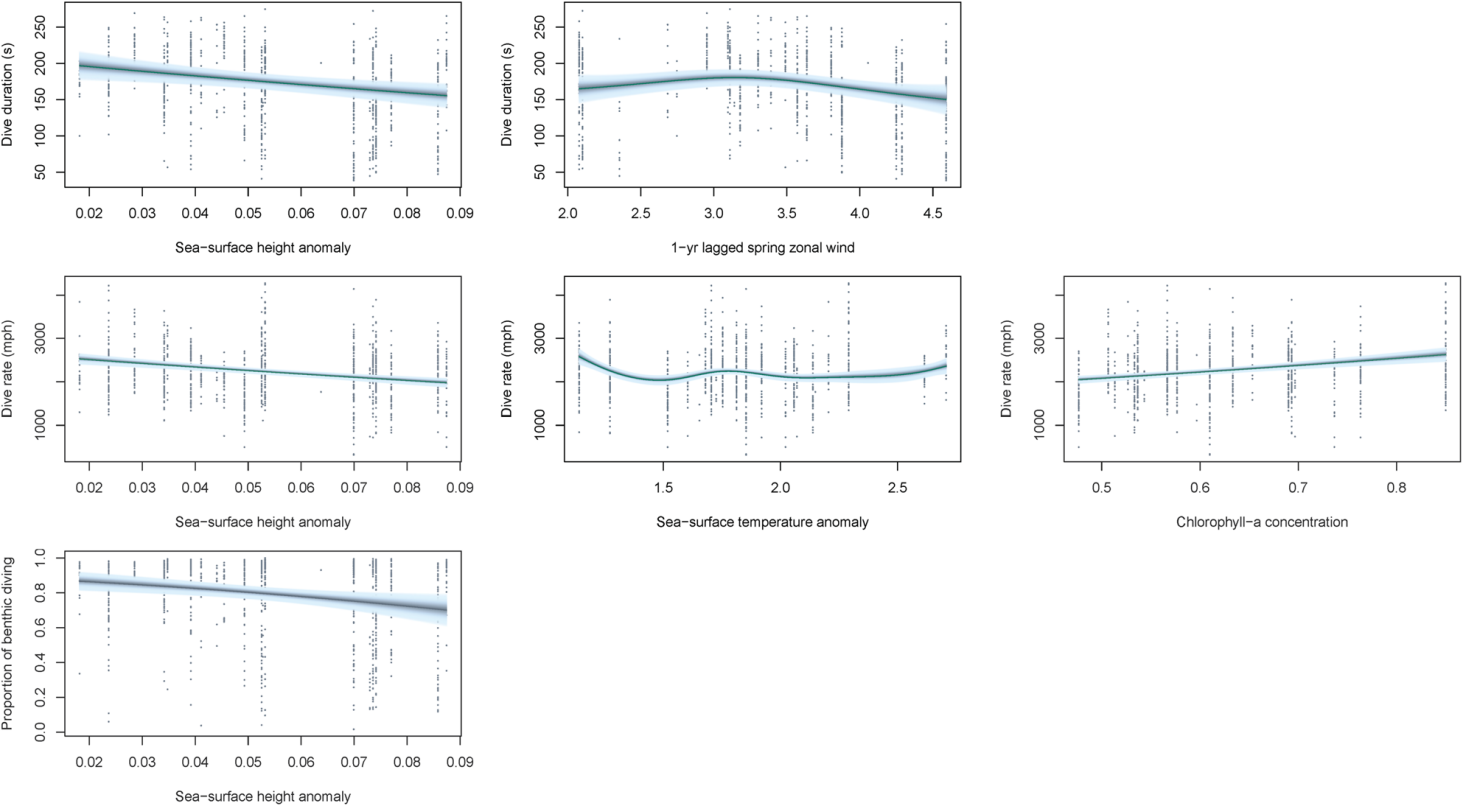
Predicted response from Generalised Additive Mixed effects Models of foraging effort of female Australian fur seals to local-scale environmental conditions. Models were constrcuted using the *mcgv* package version 1.8.31^73–73^ in the R statsitical environment version 3.6.1^51^.

**Figure 3:**
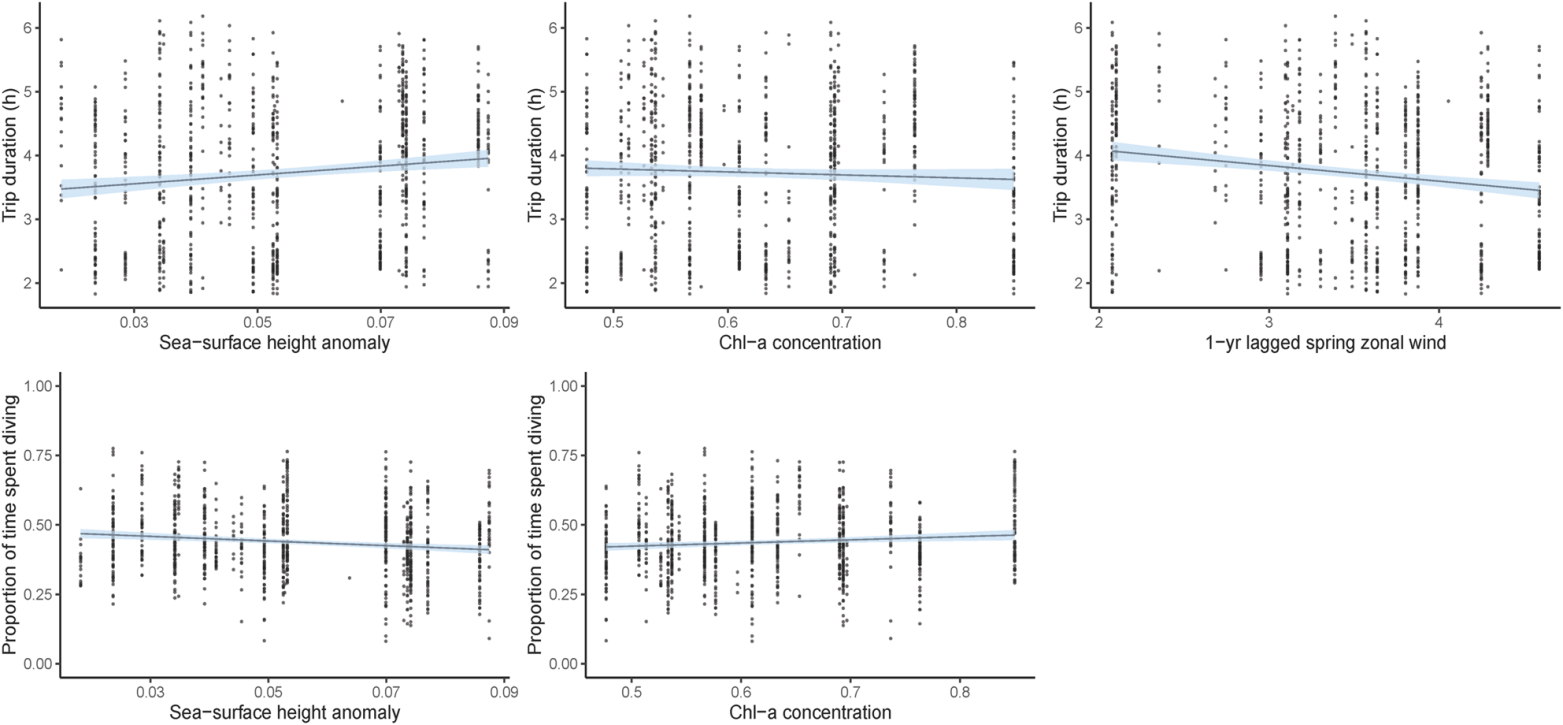
Relationships between foraging effort of female Australian fur seals and local-scale environmental conditions identified using Linear Mixed Effects models. Models were constrcuted using the *nlme* package version 3.1-140^70^ in the R statsitical environment version 3.6.1^51^.

The most parsimonious local-scale model for dive duration included current year SSHa and 1-year lagged spring zonal wind, with a significant negative influence detected for SSHa, with greater SSHa resulting in a decrease of approximately 50 s per dive (Table 2; Fig. 2). Dive rate was also found to be significantly influenced by current year SSHa, as well as current year chl-*a* concentration and SSTa (Table 2). Inspection of the smoothing parameters indicated significant increases in dive rate (approximately 700 m h^−1^ greater) with higher chl-*a* concentrations and significant decreases (approximately 250–500 m h^−1^ lower) under greater SSHa and SSTa (Table 2; Fig. 2). The most parsimonious model for trip duration included current year chl-*a*, current year SSHa and 1-year lagged spring zonal wind strength (Table 2) with significant positive effects of SSHa (approximately 20 h greater) and significant negative effects (approximately 10–30 h less) of chl-*a* and 1-year lagged zonal wind strength (Table 2; Fig. 3). Contrastingly, the proportion of time spent diving indicated a significant positive influence of chl-*a* concentration, increasing by approximately 8%, and significant negative influence of SSHa, decreasing by approximately 10% (Table 2; Fig. 3). The proportion of benthic diving was also negatively influenced by SSHa, with a decline of approximately 10% (Table 2; Fig. 3).

**Table 2:** Summary results of the Linear Mixed Effects models and Generalised Additive Mixed effects Models used to assess the effects of local-scale environmental conditions on the trip duration, benthic dive duration, benthic dive rate, proportion of time spent diving, proportion of benthic diving, Foraging Trip Success Index (FTSI) and Foraging Trip Efficiency Index (FTEI).

Response variable	Covariate	Parametric coefficients				Approximate significance of smooth terms		*p* value
		Est	SE	df	t-value	edf	F	
Dive duration (s)	(Intercept)	5.11	0.01	947	609.59			< 0.001
	IOD					3.45	10.84	< 0.001
	IOD_2_					1.00	0.02	0.901
	SAM					3.98	25.53	< 0.001
	SAM_1_					1.00	31.64	< 0.001
	SAM_2_					1.00	25.25	< 0.001
	SOI					1.00	5.97	0.015
	SOI_1_					1.00	14.29	< 0.001
	SOI_2_					1.00	0.02	0.894
Vertical dive rate (m s^−1^)	(Intercept)	7.69	0.02	947	456.46			< 0.001
	IOD					1.00	14.06	< 0.001
	IOD_2_					1.00	1.26	0.262
	SOI					1.00	0.18	0.671
Trip duration (h)	(Intercept)	3.85	0.10	828	37.94			< 0.001
	SAM_1_	0.27	0.14	828	1.89			0.059
	SOI	− 0.02	0.01	828	− 1.76			0.080
Proportion of time spent diving	(Intercept)	0.43	0.02	827	27.22			< 0.001
	IOD_2_	− 0.08	0.04	827	− 1.79			0.074
	SAM	0.03	0.02	827	1.67			0.095
	SOI_1_	0.00	0.00	827	2.42			0.016
Foraging Trip Success Index	(Intercept)	3.84	0.24	828	16.31			< 0.001
	IOD	2.19	0.71	828	3.08			0.002
	SOI_2_	0.05	0.02	828	2.98			0.003
Foraging Trip Efficiency Index	(Intercept)	0.56	0.02	829	28.88			< 0.001
	IOD_2_	0.10	0.06	829	1.58			0.115

*Est* estimated parametric coefficient, *SE* estimated standard error of parametric coefficients.

**Figure 4:**
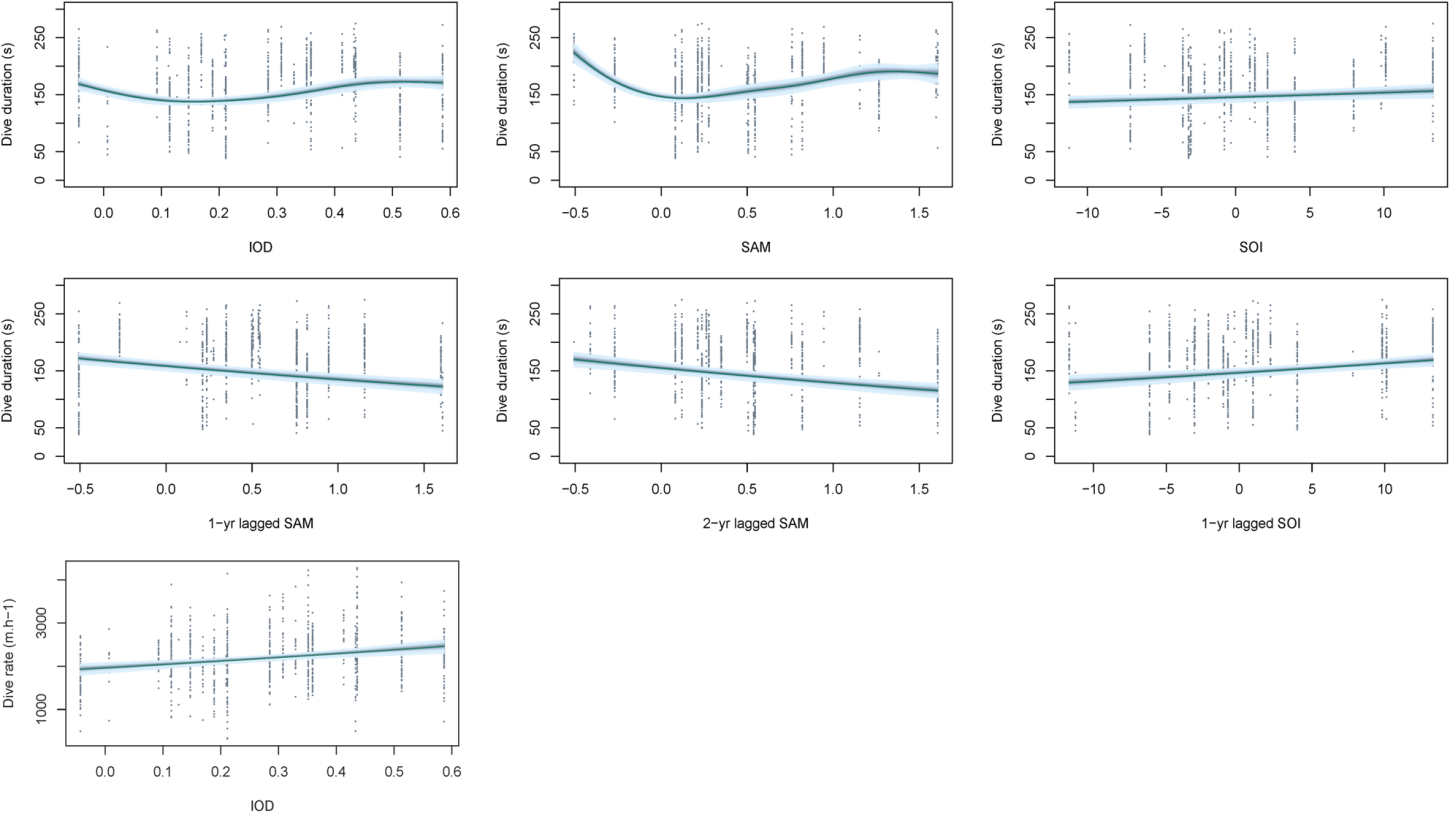
Predicted response from Generalised Additive Mixed effects Models of foraging effort of female Australian fur seals to large-scale climate indices. Models were constrcuted using the *mcgv* package version 1.8.31^71–73^ in the R statsitical environment version 3.6.1^51^.

**Figure 5:**
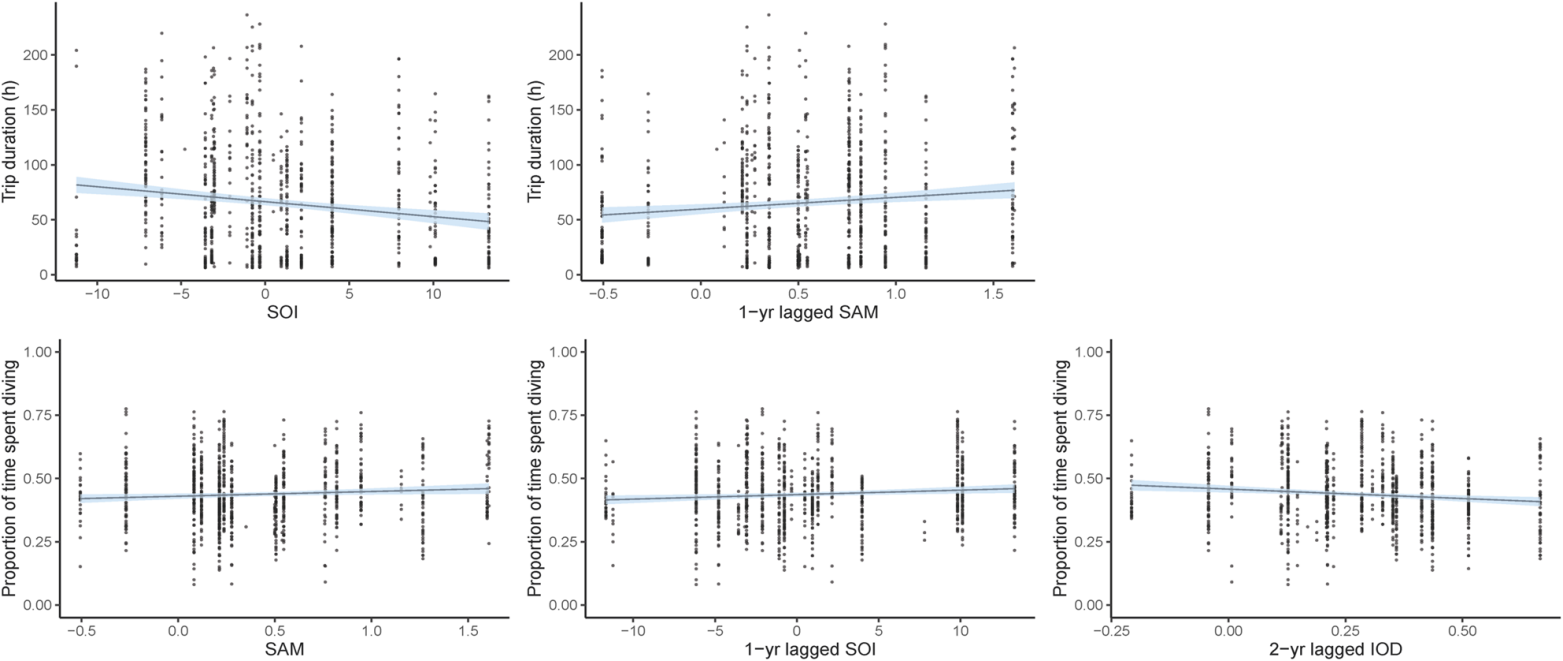
Relationships between foraging effort of female Australian fur seals and large-scale climate indices identified using Linear Mixed Effects models. Models were constrcuted using the *nlme* package version 3.1-140^70^ in the R statsitical environment version 3.6.1^51^.

The most parsimonious large-scale model for dive duration indicated significant influences of current year IOD, current year, 1-year and 2-year lagged SAM and current year and 1-year lagged SOI (Table 3). Dive duration significantly decreased, by approximately 50 s, under more positive 1-year and 2-year lagged SAM and increased, by approximately 15–35 s, under more positive current year and 1-year lagged SOI (Fig. 4). Additionally, dive duration had an overall negative influence of current year SAM, declining by approximately 30 s, while dive duration remained relatively stable under differing IOD values (Fig. 4). Benthic dive rate had a significant positive association with current year IOD, increasing by approximately 500 m h^−1^ (Table 3; Fig. 4). A positive effect of 1-year lagged SAM and negative effect of current year SOI were observed with trip duration, resulting in an increase of 50 h and decrease of 70 h, respectively (Table 3; Fig. 5). The proportion of time spent diving had a significant positive correlation with 1-year lagged SOI, increasing by approximately 6% (Table 3; Fig. 5). The most parsimonious model for the proportion of benthic diving was the null model (Table 3).

**Table 3:** Summary results of the Linear Mixed Effects models and Generalised Additive Mixed effects Models used to assess the effects of large-scale climate indices on the trip duration, benthic dive duration, benthic dive rate, proportion of time spent diving, proportion of benthic diving, Foraging Trip Success Index (FTSI) and Foraging Trip Efficiency Index (FTEI).

Response variable	Covariate	Parametric coefficients				Approximate significance of smooth terms		*p*-value
		Est	SE	df	t-value	edf	F	
Dive duration (s)	(Intercept)	5.12	0.03	947	202.38			< 0.001
	SSHa_winter_					1.00	7.27	0.007
	Wind-*u*_spring1_					2.05	2.64	0.066
Vertical dive rate (m s^−1^)	(Intercept)	7.70	0.01	947	933.30			< 0.001
	Chl-*a*_winter_					1.00	30.74	< 0.001
	SSHa_winter_					1.00	31.45	< 0.001
	SSTa_winter_					5.64	6.52	< 0.001
Trip duration (h)	(Intercept)	5.19	0.66	827	7.92			< 0.001
	Chl-*a*_winter_	− 1.69	0.83	827	− 2.05			0.041
	SSHa_winter_	11.07	4.17	827	2.65			0.008
	Wind-*u*_spring1_	− 0.22	0.11	827	− 2.09			0.037
Proportion of time spent diving	(Intercept)	0.41	0.05	828	8.87			< 0.001
	Chl-*a*_winter_	0.13	0.08	828	1.63			0.104
	SSHa_winter_	− 1.30	0.41	828	− 3.17	1.00	6.22	0.002
Proportion of benthic diving	(Intercept)	1.33	0.12	947	11.01			< 0.001
	SSHa_winter_							0.013
Foraging Trip Success Index	(Intercept)	2.93	0.70	829	4.20			< 0.001
	Chl-*a*_winter_	2.55	1.11	829	2.30			0.022
Foraging Trip Efficiency Index	(Intercept)	0.74	0.11	827	7.04			< 0.001
	Chl-*a*_winter_	− 0.21	0.13	827	− 1.56			0.120
	SSHa_winter_	1.78	0.67	827	2.66			0.008
	Wind-*u*_spring1_	− 0.04	0.02	827	− 2.06			0.039

*Est* estimated parametric coefficient, *SE* estimated standard error of parametric coefficients.

### Foraging success and efficiency

**Figure 6:**
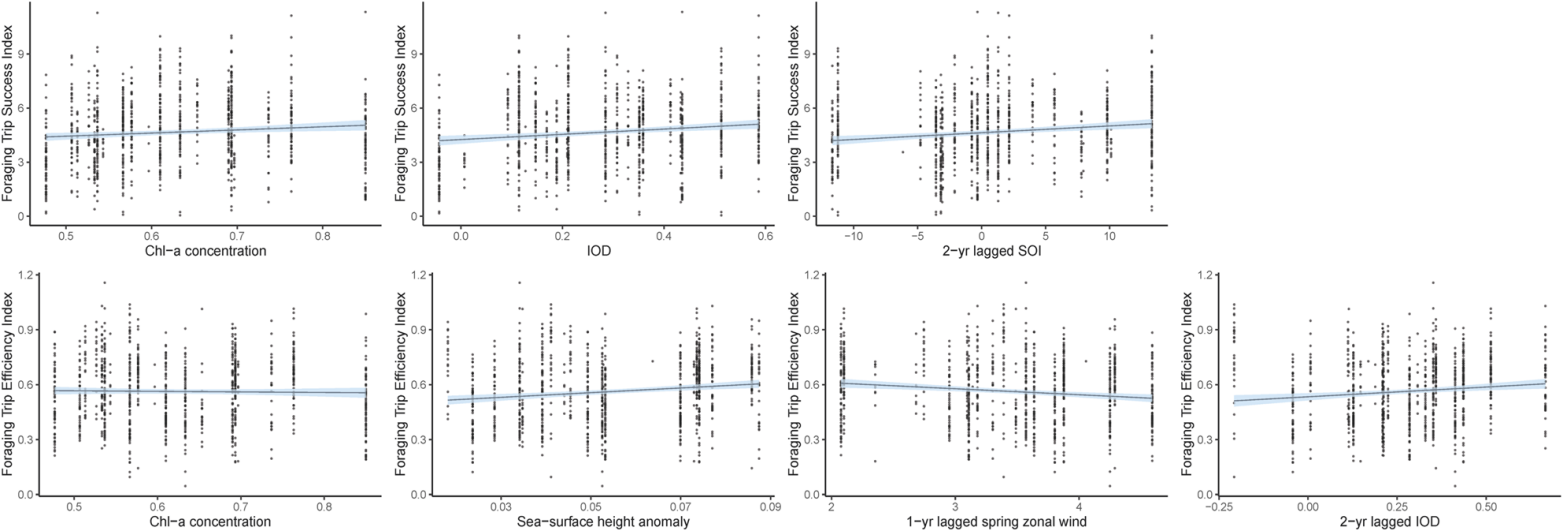
Relationships between the benthic Foraging Trip Success Index and Foraging Trip Efficiency Index of female Australian fur seals with local-scale environmental conditions and large-scale climate indices identified using Linear Mixed Effects models. Models were constrcuted using the *nlme* package version 3.1-140^70^ in the R statsitical environment version 3.6.1^51^.

When investigating the influence of local-scale environmental conditions on the foraging success and efficiency indices, chl-*a* was the only variable included in the FTSI model (Table 2). The FTSI was positively associated with current year chl-*a* (Fig. 6). Meanwhile, the most parsimonious model for FTEI included chl-*a* concentration, SSHa and 1-year lagged spring zonal wind strength (Table 2), with significant negative relationships with FTEI for chl-*a* and 1-year lagged spring zonal wind and a positive relationship with SSHa (Table 2; Fig. 6).

Current year IOD and 2-year lagged SOI were the only parameters included in the most parsimonious model investigating large-scale climate conditions on the FTSI (Table 3). The FTSI had a significant positive relationship with current year IOD and remained relatively consistent under varying 2-year lagged conditions, showing a slight downward trend (Fig. 6). The most parsimonious large-scale climate model for FTEI included 2-year lagged IOD exclusively (Table 3). However, this positive relationship was non-significant.

## Discussion

Anthropogenic environmental change has already had considerable impacts on marine ecosystems^1,76,77^, and further effects are anticipated in the coming decades as environmental change continues^9^. In the south-east Australian region, predictions indicate continued increases in sea-surface temperature and sea-surface height, weakening westerly winds and reduced primary productivity^21^. These changes will continue to have significant consequences for prey availability and distribution^22–25^, with flow on effects for top predators^26^. Therefore, understanding how marine top predators may respond to such changes is critical to predicting how their populations may respond^78,79^. The present study investigated the influence of local environmental conditions and large-scale climate indices on the foraging effort, success and efficiency of adult AUFS females provisioning pups. The results indicate complex responses to current and lagged environmental conditions. Under anticipated changes to their environment, AUFS are likely to experience further impacts on their foraging success and efficiency, which may have population-level consequences.

### Local environmental influences on foraging effort, success and efficiency

Understanding the influence of environmental conditions on foraging effort is important to elucidate their impacts on reproductive success and offspring survival in their populations^42,78,79^. Under sub-optimal foraging conditions, females provisioning offspring need to respond to changes in food availability with changes in foraging behaviour or effort^42^. However, central-place foragers are restricted in their capacity to adjust to change, leaving them particularly vulnerable to environmental change^3,4^.

The foraging behaviour and effort of individuals in the present study were significantly influenced by four local-scale environmental variables in the Bass Strait region: chl-*a* concentration, SSHa and SSTa during winter, and 1-year lagged spring zonal wind strength. None of the environmental parameters in other seasons were found to influence foraging behaviour or effort. Winter SSHa was the most influential local-scale variable on foraging effort. Winter sea-surface heights greater than average (i.e. SSHa > 0) were associated with decreases in dive rate, dive duration, the proportion of time spent diving and the proportion of benthic diving, suggesting reduced foraging effort and, potentially, improved foraging conditions or greater pelagic prey availability. However, trip duration increased with higher SSHa, which, when combined with the decrease in benthic diving, could also be suggestive of poorer foraging conditions. The increase in foraging efficiency (FTEI) under increased SSHa suggests that the former situation is more plausible.

Bass Strait in winter exhibits consistent positive sea-surface height anomalies as a result of surface intrusion of saltier and warmer South Australian Current (SAC) water into the Strait^39^. Higher sea level anomalies are indicative of increased eddy activity^80^, which is associated with increased pelagic baitfish prey abundance^56^. Indeed, an increased presence of barracouta (*Thyristes atun*), red cod (*Pseudophysis bachus*), redbait, and pilchard were observed in the diet of AUFS during years with high winter sea-surface height anomalies in the Bass Strait region^81^. Sea-surface height anomalies have also been associated with changes in foraging behaviour in southern elephant seals (*Mirounga leonina*) and New Zealand fur seals (*Arctocephalus forsteri*), likely due to increased eddy activity^82,83^.

Both the trip duration and dive duration observed in the present study decreased under stronger (i.e. more easterly or more westerly) 1-year lagged spring zonal winds in Bass Strait. This decrease in dive duration is suggestive of increased pelagic diving or increased use of shallow areas of Bass Strait, while the decreased trip duration may be indicative of individuals foraging closer to the colony. Given the lagged effect, it is possible that the stronger zonal winds alter the distribution of prey within the water column, making pelagic foraging more accessible or profitable and/or to areas nearer the colony. This is further supported by the decrease in dive duration associated with lagged SAM conditions in the present study. The SAM has a strong influence on westerly winds in southern Australia^84^, which drive nutrient rich water from the Bonney Upwelling region into Bass Strait^39,85^. Upwelling activity can greatly influence the productivity and prey availability within a system^86^, and may be driving the lagged changes in foraging effort, success and efficiency observed. However, the foraging efficiency of individuals declined with stronger 1-year lagged zonal winds, indicating that, while prey may be more readily available or accessible, individuals may have encountered greater difficulty catching or locating prey due to other factors (e.g. individual experience). Alternatively, given that the efficiency measured in the present study was for benthic foraging, individuals may have been targeting more (potentially lipid rich) pelagic prey with greater success and efficiency, which could not be captured within this study.

Whereas increased chl-*a* concentration is generally associated with increased productivity and prey availability^87,88^, the observed increases in dive rate and the proportion of time diving with higher chl-*a* concentrations would suggest poorer foraging conditions at these times^89^. However, periods of increased chl-*a* concentration in the present study also coincided with higher foraging success indices for benthic dives and reduced foraging trip durations. The co-occurrence of greater foraging effort and increased foraging success in times of higher chl-*a* concentration may reflect a higher abundance of pelagic prey in the benthic/demersal zone. Despite AUFS being predominantly benthic foragers, a considerable portion of their diet is comprised of pelagic baitfish species (e.g. redbait *Emmelichthys nitidus*, jack mackerel *Trachurus declivus* and pilchard *Sardinops sagax*^47^). While these baitfish species typically occur at depths of 40–500 m in waters beyond the continental shelf^44–46^, data from animal-borne cameras have shown that AUFS consume these species near the sea-floor within the relatively shallow (60–80 m) continental shelf of Bass Strait^90^. The smaller mass of baitfish species in comparison to other prey consumed on the benthos (e.g. octopus, elasmobranchs^91^) could necessitate individuals having a higher dive rate to meet their nutritional needs in periods of higher chl-*a* concentrations. Similarly, the greater dive durations associated with these periods could reflect individuals targeting more baitfish, which have been shown to require greater chase durations by AUFS than other demersally captured prey^91^. This would also explain the reduced foraging efficiency under higher chl-*a* conditions that was observed in the present study.

Finally, current year winter SSTa was associated with changes in dive rate, with an overall negative relationship observed. However, this relationship was more complex than other relationships discussed thus far, exhibiting fluctuations in dive rate across varying SSTa levels, initially dropping before increasing again. A similar pattern was observed for dive duration with current year SAM, and may be reflective of lower prey availability for AUFS resulting from low nutrient flow to surface waters in the Bass Strait region^58^.

### Large-scale climate influences on foraging effort, success and efficiency

In the present study, the IOD was highly influential on AUFS foraging effort, success and efficiency. Higher IOD values are associated with warmer sea surface temperatures^65^ and, in Bass Strait, winter ocean warming can result in reduced nutrient flow, reducing productivity and potentially prey availability^38^. In the present study, benthic dive rate increased with increasing IOD, while the foraging success increased under more positive current year IOD conditions. This suggests that positive IOD conditions are indicative of good foraging conditions in the same year. However, IOD is typically more influential in the spring months when IOD events peak^92^ and, thus, may impact several of the prey species consumed by AUFS that have pelagic life stages sensitive to spring environmental conditions (e.g.^14^). Indeed, the benthic foraging efficiency of female AUFS increased following 2-year lagged higher IOD, suggesting increased availability of benthic/demersal prey. While this relationship was not found to be statistically significant, the change in FTEI observed was of similar magnitude to that observed with current year SSHa and 1-year lagged zonal wind strength.

Current year and lagged SOI conditions had a strong influence on the foraging effort of female AUFS in the present study. Current year SOI was negatively correlated with trip duration and positively associated with dive duration. This increase in dive duration may be a result of reduced nutrient flow to Bass Strait surface waters during winter due to warmer surface waters and reduced ocean mixing during high SOI periods^38^. Consequently, the increase in dive duration suggests individuals spent longer periods on the sea floor searching for prey in response to reduced prey availability, shifts in prey distribution or changes in prey assemblages^93^. Alternatively, individuals may have increased dive duration to account for prey with lower lipid content, instead capturing greater quantities of lower quality prey during the dive. The latter scenario is supported by the reduction in trip duration, suggesting that individuals required shorter foraging trips under more positive SOI conditions. This may reflect the lower prey availability for AUFS resulting from low nutrient flow to surface waters in the Bass Strait region^58^. Sustained elevated negative SOI conditions are indicative of El Niño events, which are known to have strong influences on the distribution and abundance of fish species (e.g.^94^) and the foraging behaviour and success of marine top predators (e.g.^95^). Indeed, Kliska^81^ reported a positive influence of SOI on the frequency of occurrence of red cod and pilchard in the diet of AUFS, suggesting shifts in prey assemblage. Such shifts in prey assemblage likely explain the increase in the proportion of time spent diving and the dive duration observed under more positive 1-year lagged conditions.

In addition to the current year and lagged effects of SAM on the dive duration previously discussed, 1-year lagged SAM was positively correlated with trip duration. Positive SAM conditions are associated with weaker zonal winds in south-eastern Australia^96^ which can enhance the strength of the seasonal Bonney Upwelling activity to the west of Bass Strait, leading to increased productivity^58^. This can result in improved pelagic prey availability within Bass Strait^57^ in subsequent years, which is supported by the lagged reduction in dive duration observed in the present study. In this context, the increased trip duration may indicate that individuals are travelling further to reach prey patches or searching for longer for productive prey patches.

### Influence of environmental change on potential future foraging conditions

Foraging success and efficiency directly influence weaning success and subsequent offspring survival in pinnipeds^5^. As such, knowledge of the factors influencing foraging efficiency of a species is vital for predicting population level responses to environmental change. Analysis of long-term datasets on the behaviour and ecology of species can elucidate their relationships with environmental parameters which can then be used with climate forecasting to predict how species may respond to anticipated environmental change^97,98^. Many studies have reported varying population level effects of environmental change on marine mammals (reviewed^99,100^), while several studies have investigated the impacts of environmental change on foraging effort and/or efficiency of marine predators (e.g.^49,101,102^). However, few have combined these topics and projected potential population-level impacts into the future^103^, which could have substantial benefits for population management and conservation planning.

The present study has highlighted the impacts of large-scale climate indices on the foraging effort, success and efficiency of AUFS. The large-scale climate indices of IOD and SAM indicate increasingly positive phases, and this trend is predicted to continue^10,92^. These large-scale climate shifts are likely to result in further local change for south-eastern Australia, contributing to SST increases, reduced rainfall and weakening zonal winds. There is also predicted to be a shift towards more frequent and severe positive and negative ENSO events^12^ that may interact with, and exacerbate, IOD and SAM conditions^104,105^. Winter El Niño events can have a strong influence on Bonney upwelling activity and temperatures in southern Australia^85^. Each of these changes are anticipated to have substantial impacts on the distribution and abundance of prey^13^, with flow on effects to top predators in the region^106^.

The results of the present study indicate a complex array of responses in relation to current and lagged conditions, which may be changing over time. Therefore, the responses of AUFS to future conditions will depend on the magnitude of trends and on strength of inter-annual environmental fluctuations. If IOD and SAM conditions continue towards more positive phases and ENSO events become more frequent and severe, AUFS foraging effort is likely to increase to compensate for declines in prey availability. As such, sustained negative (i.e. for AUFS) environmental conditions are likely to have significant consequences for the benthic foraging success and efficiency of female AUFS, with potential consequences for pup production and offspring survival. These consequences are, in part, due to the impact of sustained high SST on productivity, prey recruitment and prey distribution^13^. However, if high magnitude conditions are infrequent, the results of the present study suggest that AUFS may benefit from lagged climate impacts through increased productivity and prey availability within Bass Strait. It is important to note that this refers only to benthic diving and may not reflect the influence of environmental change on pelagic diving effort, success or efficiency.

If local- and large-scale conditions lead to poleward shifts in prey availability outside of the Bass Strait region, AUFS may need to establish breeding colonies on offshore islands around Tasmania. As female AUFS are restricted in their foraging trip durations by provisioning pups, female AUFS may be unable to adequately provision pups if they are foraging beyond the shelf edge due to the increased travel time. Further, Bass Strait provides ideal habitat for AUFS due to their predominantly benthic foraging strategy^33^. It is likely that if AUFS established colonies beyond Bass Strait, individuals would need to target pelagic prey due to the great depths beyond the continental shelf. As such, AUFS may revert back to a pelagic foraging strategy, as seen in their conspecifics (Cape fur seals) in South Africa^43^.

In summary, the present study has highlighted the influence of local- and large-scale environmental variability on the foraging behaviour, success and efficiency of female AUFS. We infer a link to changes in prey recruitment and survival, which ultimately influence prey distribution and abundance within the region. However, an understanding of the linkages between environmental change and prey bases is lacking, which is needed to understand the mechanisms of environmental change on AUFS and other marine predators. Models with predator and prey linkages are needed to test the effects of environmental forcing that can propagate up the foodweb^107,108^. Under anticipated changes to their environment, female AUFS are likely to experience declines in foraging success and efficiency related to climate-induced shifts in prey distribution and abundance. While this study presents a reasonable prediction of how AUFS may respond to environmental change, it is uncertain how accumulative stressors may affect AUFS behaviour and distribution over time. This highlights the importance for continued monitoring of the population into the future. However, it is important to note that the indices used in the present study were calculated using data from benthic dives only and may not be reflective of pelagic diving. As such, further studies should incorporate indices based on both benthic and pelagic diving. This would provide a more complete understanding of the influence of environmental conditions on the foraging effort, success and efficiency of female AUFS and subsequent impacts on the AUFS population.

## Supplementary information

Supplementary Information
