# Author Correction: Environmental influences on foraging effort, success and efficiency in female Australian fur seals

**DOI:** 10.1038/s41598-021-96100-5

**Published:** 2021-08-12

**Authors:** Cassie N. Speakman, Andrew J. Hoskins, Mark A. Hindell, Daniel P. Costa, Jason R. Hartog, Alistair J. Hobday, John P. Y. Arnould

**Affiliations:** 1 School of Life and Environmental Sciences, Deakin University, Burwood, VIC Australia; 2 CSIRO Health and Biosecurity, Townsville, QLD Australia; 3 Institute for Marine and Antarctic Studies, University of Tasmania, Hobart, TAS Australia; 4 Ecology and Evolutionary Biology Department, University of California Santa Cruz, Santa Cruz, CA USA; 5 CSIRO Oceans and Atmosphere, Hobart, TAS Australia

Correction to: *Scientific Reports* 10.1038/s41598-020-73579-y, published online 19 October 2020

The original version of this Article contained an error.

In the Discussion section, subheading “Large-scale climate influences on foraging effort, success and efficiency”,

“Sustained elevated positive SOI conditions are indicative of El Niño events, which are known to have strong influences on the distribution and abundance of fish species (e.g.^94^) and the foraging behaviour and success of marine top predators (e.g.^95^).”

now reads:

“Sustained elevated negative SOI conditions are indicative of El Niño events, which are known to have strong influences on the distribution and abundance of fish species (e.g.^94^) and the foraging behaviour and success of marine top predators (e.g.^95^).”

The original Article has been corrected.

