# Supplementary material for: Environmental influences on foraging effort, success and efficiency in female Australian fur seals
Source: Sci Rep. 2020 Oct 19;10:17710. doi: 10.1038/s41598-020-73579-y (PMC7572486; doi:10.1038/s41598-020-73579-y)
Supplement: Supplementary file 1 — Supplementary Information [file 41598_2020_73579_MOESM1_ESM.pdf]

Environmental influences on foraging effort, success and efficiency in female Australian fur seals

Cassie N. Speakman[1], Andrew J. Hoskins[2], Mark A. Hindell[3], Daniel P. Costa[4], Jason R. Hartog[5], Alistair J. Hobday[5] and John P.Y. Arnould[1]

[1]Deakin University, School of Life and Environmental Sciences, Burwood, Victoria, Australia

[2]CSIRO Health and Biosecurity, Townsville, Queensland, Australia

[3]Institute for Marine and Antarctic Studies, University of Tasmania, Hobart, Tasmania, Australia

[4]Ecology and Evolutionary Biology Department, University of California Santa Cruz, Santa Cruz, California, USA

[5]CSIRO Oceans and Atmosphere, Hobart, Tasmania, Australia

Supplementary table S1. Example of classification of dive types using the bimodal distribution of the kernel density estimate dives. Points falling to the right of the nadir (red line) are classified as benthic, whereas dives to the left of the nadir are classified as pelagic. The quantity of each dive type is indicated in the 'Example trip' column, whereby Pel = pelagic and Ben = benthic.

<table>
<tr><th>Example trip</th><th>Bimodal distribution of dives</th><th>Frequency of dives in each peak</th></tr>
<tr><td>1
Pel: 68
Ben: 252</td><td>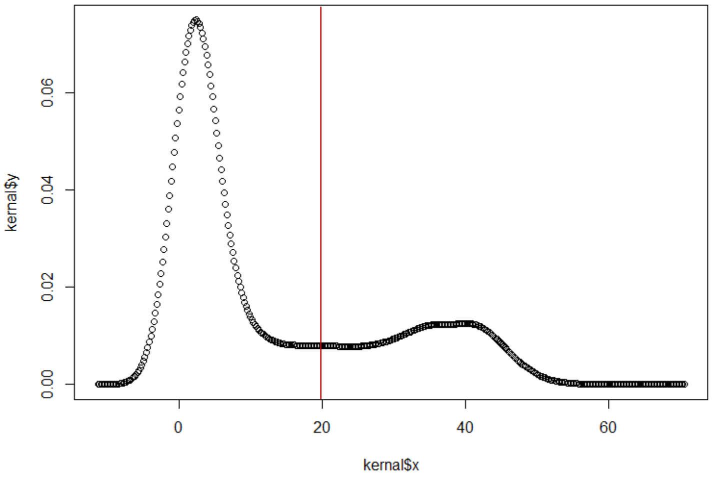
</td><td>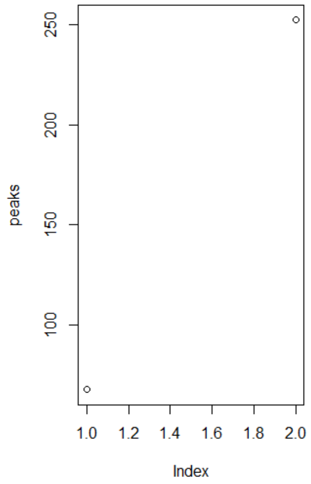
</td></tr>
<tr><td>2
Pel: 68
Ben: 287</td><td>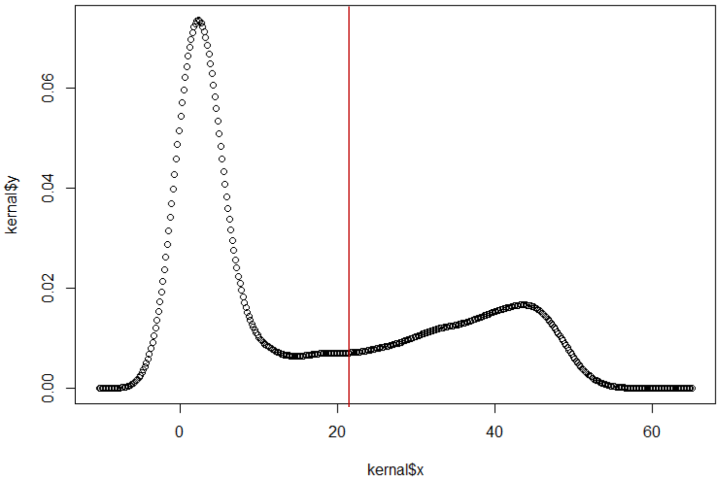
</td><td>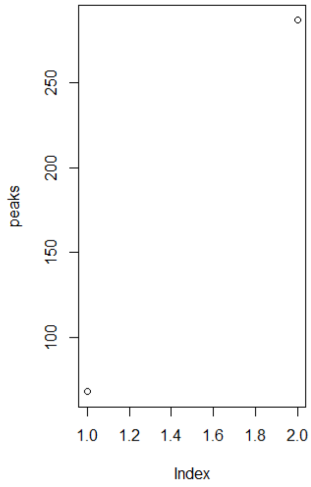
</td></tr>
<tr><td>3
Pel: 61
Ben: 270</td><td>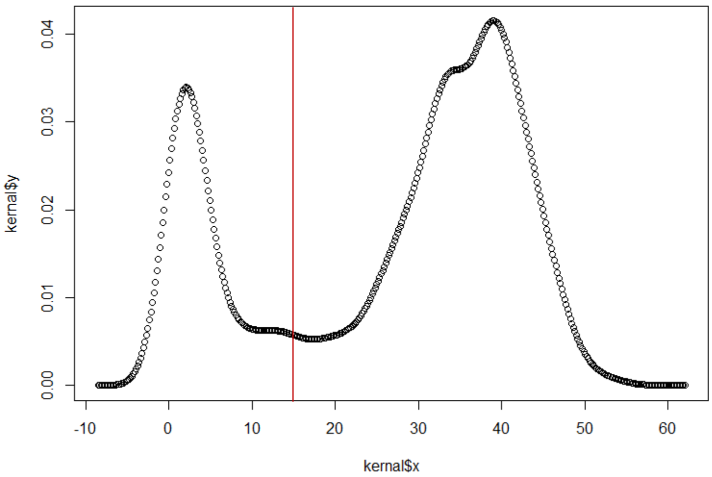
</td><td>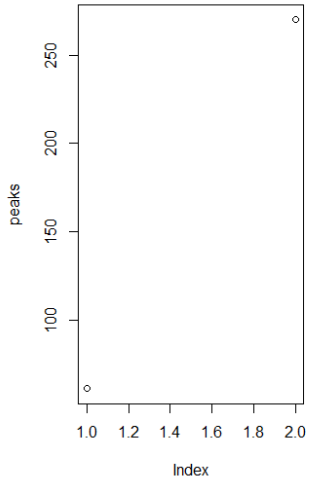
</td></tr>
</table>

Supplementary table S2. Dive metrics for individual Australian fur seals (*Arctocephalus pusillus doriferus*) from Kanowna Island. *Individual sampled in two separate years.

Seal ID	Year	Deployment duration (d)	No. of trips analysed	No. of dives analysed	Trip duration (d)	Dive duration (min)	Proportion of time spent diving (%)	Max depth (m)	Descent rate (m s-1)	Ascent rate (m s-1)	Dive rate (m h-1)	Proportion of benthic diving (%)	Foraging Trip Success Index	Foraging Trip Efficiency Index
1	1998	2.8	1	157	1.06	3.57	36.66	82.00	0.71	1.48	32.96	3.80	3.32	0.00
2	1998	121.2	9	9620	5.54 ± 0.25	2.82 ± 0.06	37.65 ± 2.30	85.33 ± 0.24	1.34 ± 0.08	1.38 ± 0.11	1044.06 ± 27.85	85.9 ± 1.7	14.22 ± 0.52	0.22 ± 0.02
3	1998	102.2	9	11788	7.35 ± 0.46	3.10 ± 0.07	38.57 ± 3.52	83.78 ± 0.24	1.66 ± 0.08	1.75 ± 0.02	1108.54 ± 29.42	97.1 ± 0.5	15.52 ± 0.20	0.36 ± 0.02
4	1998	8.1	2	1467	2.18 ± 1.88	2.23 ± 0.39	46.40 ± 3.84	81.50 ± 0.24	0.94 ± 0.08	0.81 ± 0.08	912.64 ± 305.62	69.9 ± 21.2	11.22 ± 0.29	0.07 ± 0.06
5	1998	5.0	1	619	3.65	3.58	42.13	83.00	1.30	1.41	1004.83	88.40	8.40	0.12
6	1998	6.6	2	1215	3.22 ± 1.67	3.98 ± 0.35	47.64 ± 4.46	83.50 ± 0.24	1.15 ± 0.08	1.30 ± 0.04	808.77 ± 104.82	89.9 ± 0.9	5.22 ± 0.67	0.11 ± 0.06
7	1998	8.2	4	1417	1.56 ± 0.50	3.75 ± 0.46	55.57 ± 9.35	78.75 ± 0.24	1.12 ± 0.08	1.15 ± 0.16	951.59 ± 104.32	81.3 ± 10.4	6.37 ± 0.55	0.07 ± 0.02
8	1999	125.4	4	4295	6.27 ± 0.30	3.16 ± 0.08	37.67 ± 6.23	85.75 ± 0.24	1.60 ± 0.08	1.70 ± 0.02	1120.24 ± 98.85	94.9 ± 2.8	14.17 ± 0.40	0.26 ± 0.04
9	1999	77.1	14	6423	3.37 ± 0.55	3.09 ± 0.08	30.16 ± 8.11	74.54 ± 0.24	1.22 ± 0.08	1.36 ± 0.09	625.90 ± 57.50	87.0 ± 2.3	8.88 ± 0.48	0.10 ± 0.02
10	1999	76.0	10	9808	4.99 ± 0.58	2.35 ± 0.12	31.27 ± 8.36	82.30 ± 0.24	1.08 ± 0.08	1.06 ± 0.08	522.56 ± 28.94	47.0 ± 2.6	8.38 ± 0.43	0.08 ± 0.01
11	1999	65.5	19	12082	1.68 ± 0.23	2.38 ± 0.13	54.21 ± 8.95	66.42 ± 0.24	1.14 ± 0.08	1.12 ± 0.07	1038.37 ± 44.30	90.1 ± 3.0	8.24 ± 0.49	0.11 ± 0.03
12	2001	13.7	3	1862	3.76 ± 0.69	2.62 ± 0.22	29.06 ± 3.74	119.33 ± 0.24	1.39 ± 0.08	1.54 ± 0.15	629.55 ± 90.72	57.2 ± 8.6	9.01 ± 1.52	0.08 ± 0.02
13	2002	69.2	14	7166	3.25 ± 0.58	3.58 ± 0.14	40.89 ± 9.70	82.11 ± 0.24	1.28 ± 0.08	1.35 ± 0.06	872.40 ± 53.85	85.6 ± 2.7	7.33 ± 0.37	0.10 ± 0.02
14	2002	9.3	2	1575	4.46 ± 1.54	2.52 ± 0.20	32.23 ± 7.92	85.50 ± 0.24	1.16 ± 0.08	1.00 ± 0.01	789.08 ± 82.13	65.2 ± 0.3	12.02 ± 1.29	0.10 ± 0.03
15	2002	66.3	9	8374	5.15 ± 0.80	3.85 ± 0.13	47.32 ± 5.90	84.22 ± 0.24	1.38 ± 0.08	1.24 ± 0.06	1028.88 ± 50.54	88.6 ± 1.7	9.00 ± 0.53	0.24 ± 0.04
16	2002	11.6	1	238	1.85	3.50	31.28	81.50	1.01	1.02	620.38	73.50	6.44	0.04
17	2003	59.0	41	8140	0.79 ± 0.06	2.72 ± 1.03	49.88 ± 9.51	63.22 ± 1.24	1.38 ± 0.08	1.57 ± 0.02	1195.59 ± 34.65	95.2 ± 0.4	12.75 ± 0.17	0.05 ± 1.00
18	2003	88.4	35	23466	1.69 ± 0.17	1.55 ± 1.12	44.47 ± 18.57	56.17 ± 1.24	1.01 ± 0.08	0.95 ± 0.05	580.12 ± 46.36	55.6 ± 4.4	8.09 ± 0.51	0.07 ± 1.01
19	2003	66.1	9	8038	5.76 ± 0.44	3.72 ± 1.08	39.92 ± 4.91	85.61 ± 1.24	1.38 ± 0.08	1.29 ± 0.02	947.38 ± 35.67	93.0 ± 1.1	9.37 ± 0.41	0.20 ± 1.02
20	2003	12.3	3	805	2.09 ± 0.60	2.81 ± 1.36	24.91 ± 5.71	80.83 ± 1.24	1.00 ± 0.08	0.94 ± 0.22	627.10 ± 89.90	76.5 ± 6.8	10.25 ± 0.84	0.05 ± 1.02
21	2003	11.0	2	944	2.73 ± 0.11	3.16 ± 1.18	38.19 ± 6.36	83.75 ± 1.24	1.05 ± 0.08	1.04 ± 0.13	839.87 ± 85.86	73.0 ± 3.9	7.35 ± 0.12	0.07 ± 1.00
22	2003	6.7	2	791	2.24 ± 0.54	3.40 ± 1.05	41.56 ± 0.06	82.25 ± 1.24	1.43 ± 0.08	1.54 ± 0.02	1061.95 ± 6.34	93.3 ± 0.6	9.88 ± 0.71	0.08 ± 1.01
23	2003	10.4	4	796	1.03 ± 0.26	2.84 ± 1.32	38.69 ± 7.53	81.50 ± 1.24	1.09 ± 0.08	1.09 ± 0.22	933.94 ± 71.84	74.0 ± 8.2	11.05 ± 1.77	0.04 ± 1.01
24	2004	8.7	1	634	4.76	3.34	30.95	84.00	1.43	1.49	833.89	93.10	10.55	0.13
25	2010	12.0	3	1258	2.54 ± 0.39	4.18 ± 1.04	48.19 ± 2.28	83.50 ± 1.24	1.40 ± 0.08	1.51 ± 0.02	998.50 ± 4.53	89.8 ± 1.4	6.72 ± 0.29	0.09 ± 1.01
26	2005	18.1	7	2894	1.51 ± 0.42	2.30 ± 1.21	45.92 ± 4.58	66.71 ± 1.24	1.15 ± 0.08	0.98 ± 0.07	855.79 ± 75.56	95.3 ± 1.2	7.16 ± 1.54	0.05 ± 1.02
27	2005	44.0	19	4982	1.24 ± 0.17	2.82 ± 1.17	43.89 ± 12.15	66.79 ± 1.24	1.10 ± 0.08	1.17 ± 0.05	799.05 ± 48.14	91.1 ± 1.8	7.97 ± 0.81	0.05 ± 1.01
28	2005	53.4	12	4225	3.00 ± 0.57	3.31 ± 1.17	30.49 ± 11.47	140.29 ± 1.24	1.11 ± 0.08	1.09 ± 0.05	673.17 ± 80.54	64.9 ± 4.6	5.06 ± 0.45	0.03 ± 1.01
29	2005	44.1	7	4894	4.39 ± 0.56	3.38 ± 1.09	39.05 ± 8.59	85.29 ± 1.24	1.43 ± 0.08	1.59 ± 0.04	1018.83 ± 93.38	91.1 ± 2.9	10.22 ± 0.52	0.14 ± 1.01
30	2005	101.5	17	9123	2.56 ± 0.64	2.90 ± 1.20	38.08 ± 8.99	82.29 ± 1.24	1.09 ± 0.08	1.07 ± 0.08	876.16 ± 54.71	72.4 ± 5.1	9.34 ± 0.76	0.08 ± 1.02
31	2006	5.1	1	356	2.97	3.39	28.14	84.50	0.96	0.88	550.02	67.70	5.40	0.04

32	2006	46.3	10	6159	3.18 ± 0.50	2.78 ± 1.05	39.32 ± 9.74	82.65 ± 1.24	1.70 ± 0.08	1.62 ± 0.01	1269.20 ± 81.59	94.6 ± 0.7	17.31 ± 0.50	0.14 ± 1.02
33	2006	5.7	1	715	3.96	3.16	39.67	86.00	0.75	0.75	969.57	77.10	8.78	0.12
34	2006	40.6	5	5164	6.38 ± 0.57	2.89 ± 1.08	32.53 ± 4.29	85.30 ± 1.24	1.32 ± 0.08	1.43 ± 0.19	953.13 ± 41.12	89.3 ± 3.1	12.48 ± 0.77	0.19 ± 1.03
35	2006	3.6	1	607	1.83	1.67	38.48	85.50	0.68	0.80	757.4	33.60	7.80	0.04
36	2007	62.1	25	5097	1.13 ± 0.17	3.29 ± 1.07	46.40 ± 10.85	79.56 ± 1.24	1.16 ± 0.08	1.25 ± 0.07	1049.73 ± 54.24	85.5 ± 2.0	8.93 ± 0.27	0.04 ± 1.00
37	2007	9.5	4	1466	1.67 ± 0.42	2.44 ± 1.15	32.82 ± 6.70	81.38 ± 1.24	1.05 ± 0.08	0.95 ± 0.10	677.64 ± 116.99	65.0 ± 3.3	6.55 ± 1.65	0.04 ± 1.02
38	2007	53.1	9	7739	3.94 ± 0.38	3.19 ± 1.15	50.60 ± 16.48	85.17 ± 1.24	1.24 ± 0.08	1.26 ± 0.13	1288.01 ± 147.13	84.9 ± 3.6	8.85 ± 1.28	0.14 ± 1.03
39	2007	51.9	15	6386	2.07 ± 0.49	2.77 ± 1.11	45.15 ± 13.62	83.30 ± 1.24	0.99 ± 0.08	1.03 ± 0.08	1144.64 ± 113.84	75.2 ± 4.8	9.92 ± 0.45	0.06 ± 1.01
40	2007	64.9	15	8239	2.76 ± 0.35	3.00 ± 1.12	41.12 ± 7.22	82.37 ± 1.24	1.18 ± 0.08	1.14 ± 0.07	803.35 ± 96.00	63.7 ± 7.0	6.52 ± 0.70	0.07 ± 1.01
41	2007	3.7	1	553	3.71	4.15	43.01	85.00	0.92	0.89	903.68	92.90	4.74	0.08
42	2007	4.7	2	1040	2.24 ± 1.52	3.64 ± 1.48	59.27 ± 12.02	85.75 ± 1.24	1.00 ± 0.08	0.98 ± 0.15	1093.11 ± 91.99	95.4 ± 2.9	5.66 ± 0.94	0.09 ± 1.06
43	2008	10.9	2	1188	3.32 ± 1.00	3.56 ± 1.23	44.69 ± 6.08	86.00 ± 1.24	1.18 ± 0.08	1.34 ± 0.12	1020.80 ± 36.16	85.0 ± 0.0	6.09 ± 1.95	0.08 ± 1.04
44	2008	8.0	1	571	3.16	3.24	40.66	87.00	1.36	1.47	1095.58	88.10	9.78	0.10
45	2008	26.5	2	256	0.47 ± 0.11	3.16 ± 1.23	60.83 ± 12.49	77.25 ± 1.24	1.33 ± 0.08	1.41 ± 0.01	1300.60 ± 198.02	84.0 ± 0.1	8.35 ± 1.22	0.02 ± 1.00
46	2008	4.1	1	365	2.46	3.88	39.91	81.00	1.32	1.18	869.09	89.00	7.71	0.08
47	2008	10.3	3	959	1.76 ± 0.67	3.97 ± 1.10	49.40 ± 1.85	81.83 ± 1.24	1.21 ± 0.08	1.33 ± 0.02	979.63 ± 29.75	84.4 ± 1.4	5.25 ± 0.18	0.05 ± 1.02
48	2008	14.2	2	1725	5.79 ± 0.37	3.85 ± 1.03	39.88 ± 0.92	82.50 ± 1.24	1.19 ± 0.08	1.39 ± 0.08	854.11 ± 96.03	90.3 ± 7.0	6.12 ± 1.36	0.14 ± 1.03
49	2008	42.6	17	3764	1.22 ± 0.25	3.45 ± 1.06	50.01 ± 10.85	82.41 ± 1.24	1.52 ± 0.08	1.41 ± 0.02	1260.66 ± 73.06	90.2 ± 0.9	11.76 ± 0.54	0.05 ± 1.01
50	2008	43.2	8	4849	3.64 ± 0.63	3.93 ± 1.13	47.07 ± 7.08	87.69 ± 1.24	1.42 ± 0.08	1.41 ± 0.02	1072.90 ± 53.08	91.5 ± 1.8	8.85 ± 0.57	0.14 ± 1.02
51	2008	5.8	5	868	0.54 ± 0.03	3.14 ± 1.07	69.43 ± 4.25	56.20 ± 1.24	1.21 ± 0.08	1.44 ± 0.04	1021.47 ± 41.99	91.3 ± 2.9	8.20 ± 0.45	0.05 ± 1.01
52	2008	6.6	1	646	3.87	2.76	31.93	87.00	1.76	1.57	1102.22	95.40	17.95	0.15
53	2008	5.9	1	710	4.06	3.07	37.31	83.00	1.60	1.49	1094.92	96.20	14.50	0.18
54	2009	12.5	4	1005	1.28 ± 0.38	3.28 ± 1.15	48.74 ± 7.93	80.38 ± 1.24	1.36 ± 0.08	1.45 ± 0.05	1229.07 ± 112.63	91.6 ± 1.9	9.29 ± 0.42	0.05 ± 1.01
55	2009	9.8	3	1733	3.03 ± 1.58	3.05 ± 1.09	34.34 ± 9.77	81.17 ± 1.24	1.24 ± 0.08	1.25 ± 0.02	674.62 ± 105.72	87.2 ± 3.2	8.14 ± 0.53	0.12 ± 1.08
56	2009	7.7	3	1096	1.65 ± 0.62	3.22 ± 1.22	55.77 ± 13.15	74.17 ± 1.24	1.43 ± 0.08	1.53 ± 0.03	1220.88 ± 111.06	90.6 ± 3.9	10.24 ± 1.09	0.09 ± 1.02
57	2009	17.3	7	2724	1.60 ± 0.38	2.35 ± 1.17	44.17 ± 5.27	65.29 ± 1.24	1.27 ± 0.08	1.32 ± 0.13	1043.66 ± 48.41	86.2 ± 2.7	12.51 ± 0.86	0.08 ± 1.02
58	2009	38.6	8	5295	3.59 ± 0.96	2.51 ± 1.08	34.63 ± 8.44	95.88 ± 1.24	0.92 ± 0.08	0.77 ± 0.02	883.69 ± 58.83	75.5 ± 1.9	9.23 ± 0.96	0.09 ± 1.02
59	2009	40.3	6	6321	4.67 ± 0.65	2.59 ± 1.09	41.44 ± 6.74	83.50 ± 1.24	1.58 ± 0.08	1.48 ± 0.09	1300.02 ± 95.80	84.5 ± 4.4	17.88 ± 0.44	0.22 ± 1.03
60	2009	5.6	1	570	3.29	2.77	33.23	83.50	1.04	0.92	810.89	72.30	9.27	0.08
61	2009	4.0	1	708	3.71	3.57	47.30	84.50	1.10	1.19	1024.46	81.80	5.87	0.09
62	2009	4.4	1	561	3.14	2.86	35.48	84.50	0.88	0.80	928.02	76.50	6.48	0.06
63	2009	8.3	1	1036	7.35	3.46	33.87	87.00	1.11	1.38	833.8	86.00	5.72	0.11
64	2009	5.7	1	657	4.24	3.31	35.60	84.50	1.51	1.44	1000.67	94.80	11.89	0.15
65	2009	5.9	1	1044	5.43	3.07	41.02	83.00	1.48	1.71	1258.32	98.60	13.00	0.22
66	2009	10.6	1	2328	9.5	2.31	39.23	105.00	1.14	1.02	943	62.90	12.38	0.28
67	2009	30.2	16	4384	1.08 ± 0.20	2.62 ± 1.12	47.37 ± 8.40	68.72 ± 1.24	1.19 ± 0.08	1.21 ± 0.09	1077.07 ± 61.60	82.3 ± 4.1	11.35 ± 0.57	0.05 ± 1.01
68	2009	10.5	6	1718	1.21 ± 0.39	2.76 ± 1.24	51.75 ± 14.36	79.83 ± 1.24	1.03 ± 0.08	1.01 ± 0.20	1283.71 ± 136.68	80.1 ± 5.1	9.51 ± 0.50	0.05 ± 1.01
69	2009	9.2	3	1932	2.07 ± 1.33	2.96 ± 1.44	51.34 ± 6.23	62.33 ± 1.24	1.10 ± 0.08	1.28 ± 0.07	740.33 ± 127.60	87.1 ± 4.2	5.59 ± 1.34	0.14 ± 1.11

70	2009	23.7	4	2810	4.58 ± 1.18	4.22 ± 1.09	45.02 ± 11.09	80.50 ± 1.24	1.18 ± 0.08	1.19 ± 0.02	795.70 ± 119.14	89.3 ± 5.0	3.80 ± 0.54	0.11 ± 1.03
71	2009	140.5	7	9715	6.12 ± 0.99	2.86 ± 1.17	43.17 ± 6.08	85.71 ± 1.24	1.14 ± 0.08	1.11 ± 0.09	984.71 ± 86.16	73.4 ± 7.4	10.72 ± 0.71	0.21 ± 1.03
72	2010	6.7	1	999	5.87	3.34	39.50	85.50	1.47	1.45	1093.8	94.20	11.82	0.23
73	2010	6.4	1	640	3.71	3.73	44.68	83.00	1.38	1.53	1092.99	95.60	8.96	0.14
74	2010	17.2	4	2514	2.50 ± 0.87	2.30 ± 1.12	42.55 ± 8.47	64.88 ± 1.24	1.11 ± 0.08	0.98 ± 0.08	676.25 ± 26.31	71.5 ± 9.5	8.67 ± 0.98	0.08 ± 1.02
75	2011	30.0	18	5661	0.88 ± 0.17	2.76 ± 1.24	55.17 ± 13.11	77.17 ± 1.24	1.10 ± 0.08	0.98 ± 0.05	875.44 ± 79.35	58.8 ± 6.3	4.05 ± 0.71	0.02 ± 1.00
76	2011	6.5	2	776	1.82 ± 0.47	2.50 ± 1.63	31.39 ± 3.17	83.00 ± 1.24	1.10 ± 0.08	1.07 ± 0.27	770.09 ± 31.54	70.9 ± 11.9	12.08 ± 1.82	0.06 ± 1.02
77	2011	29.7	8	3593	2.19 ± 0.41	2.88 ± 1.09	45.36 ± 11.01	64.25 ± 1.24	1.31 ± 0.08	1.37 ± 0.03	847.74 ± 43.21	93.1 ± 2.0	9.42 ± 0.33	0.10 ± 1.01
78	2011	4.3	1	656	3.33	2.94	40.28	86.00	1.55	1.45	1282.82	94.50	14.76	0.14
79	2011	20.4	4	2345	3.74 ± 1.07	3.71 ± 1.06	40.98 ± 4.27	84.13 ± 1.24	1.34 ± 0.08	1.41 ± 0.04	963.05 ± 51.74	91.7 ± 2.5	8.65 ± 0.42	0.12 ± 1.03
80*	2011	3.9	1	511	2.60	3.22	44.03	83.5	1.46	1.41	1134.25	87.7	12.04	0.11
80*	2018	7.9	2	980	3.01 ± 0.23	4.07 ± 0.19	46.03 ± 1.51	88.00 ± 0.00	1.27 ± 0.01	1.17 ± 0.03	1010.38 ± 44.07	90.8 ± 5.2	6.70 ± 0.41	0.1 ± 0.01
81	2011	6.0	1	556	4.04	3.70	35.33	86.00	1.35	1.52	908.49	94.60	8.66	0.11
82	2019	75.4	16	9440	3.40 ± 0.52	3.11 ± 1.16	36.36 ± 8.39	78.78 ± 1.24	1.36 ± 0.08	1.43 ± 0.04	757.01 ± 72.50	76.9 ± 5.7	8.97 ± 0.59	0.09 ± 1.02
83	2011	40.9	5	5129	5.78 ± 0.40	3.44 ± 1.13	42.33 ± 7.67	86.40 ± 1.24	1.39 ± 0.08	1.43 ± 0.15	1136.22 ± 118.98	91.7 ± 5.3	11.60 ± 0.59	0.25 ± 1.04
84	2011	37.6	23	4486	0.95 ± 0.20	3.40 ± 1.08	57.30 ± 14.35	81.00 ± 1.24	1.35 ± 0.08	1.34 ± 0.05	1454.34 ± 94.72	93.7 ± 1.1	10.96 ± 0.56	0.04 ± 1.01
85	2011	40.4	11	2474	1.30 ± 0.15	3.28 ± 1.03	40.71 ± 6.06	81.77 ± 1.24	1.46 ± 0.08	1.43 ± 0.02	1135.70 ± 46.83	95.8 ± 0.9	12.03 ± 0.55	0.05 ± 1.01
86	2012	31.3	5	4029	4.15 ± 0.26	3.30 ± 1.06	44.47 ± 0.89	84.90 ± 1.24	1.53 ± 0.08	1.62 ± 0.03	1274.63 ± 12.98	96.3 ± 0.9	12.83 ± 0.34	0.19 ± 1.01
87	2012	16.6	4	2102	2.67 ± 0.28	3.09 ± 1.17	42.56 ± 5.34	80.50 ± 1.24	1.30 ± 0.08	1.37 ± 0.24	1123.83 ± 88.49	93.6 ± 3.8	12.80 ± 0.29	0.13 ± 1.02
88	2012	35.1	6	7289	4.05 ± 1.50	2.43 ± 1.40	39.27 ± 8.74	72.50 ± 1.24	0.87 ± 0.08	0.83 ± 0.08	746.49 ± 51.56	68.0 ± 9.4	10.29 ± 1.37	0.15 ± 1.05
89	2012	13.9	7	2896	1.79 ± 0.34	3.31 ± 1.19	53.00 ± 3.22	84.07 ± 1.24	1.11 ± 0.08	1.07 ± 0.13	1276.57 ± 41.36	83.7 ± 4.1	10.51 ± 0.31	0.09 ± 1.02
90	2012	5.6	1	814	5.64	3.99	40.02	86.00	1.37	1.30	939.61	95.70	8.39	0.19
91	2012	19.0	6	1381	1.38 ± 0.52	3.67 ± 1.04	48.16 ± 9.01	77.08 ± 1.24	1.40 ± 0.08	1.42 ± 0.01	1122.05 ± 73.62	95.9 ± 1.1	9.75 ± 0.30	0.06 ± 1.02
92	2012	36.3	5	4486	4.73 ± 0.84	3.20 ± 1.07	42.83 ± 3.29	85.80 ± 1.24	1.48 ± 0.08	1.54 ± 0.02	1212.53 ± 41.89	92.3 ± 3.4	12.24 ± 0.38	0.20 ± 1.03
93	2012	13.3	3	1639	3.29 ± 1.68	3.77 ± 1.06	46.48 ± 5.90	84.00 ± 1.24	1.33 ± 0.08	1.35 ± 0.03	1115.98 ± 103.32	93.5 ± 2.1	8.30 ± 0.75	0.12 ± 1.06
94	2012	8.6	5	711	0.81 ± 0.16	3.91 ± 1.10	49.71 ± 12.32	71.40 ± 1.24	1.24 ± 0.08	1.36 ± 0.02	923.70 ± 105.54	93.5 ± 2.3	6.94 ± 0.35	0.03 ± 1.01
95	2012	10.3	3	1342	1.75 ± 0.18	3.68 ± 1.14	65.23 ± 6.40	70.83 ± 1.24	1.35 ± 0.08	1.51 ± 0.01	1176.18 ± 69.85	92.8 ± 3.7	8.23 ± 0.88	0.12 ± 1.02
96	2012	9.0	4	1258	1.34 ± 0.52	2.92 ± 1.42	43.78 ± 8.10	79.13 ± 1.24	0.90 ± 0.08	0.92 ± 0.16	1019.94 ± 73.55	74.1 ± 7.7	10.44 ± 0.61	0.05 ± 1.02
97	2012	2.7	1	480	2.7	3.31	40.84	82.00	1.61	1.56	1162.59	98.50	14.28	0.13
98	2012	26.4	6	6429	3.05 ± 1.18	1.34 ± 1.21	31.89 ± 7.64	69.33 ± 1.24	0.92 ± 0.08	0.80 ± 0.06	622.17 ± 73.92	45.0 ± 8.3	8.79 ± 0.97	0.04 ± 1.01
99	2012	8.6	1	1145	6.53	3.81	46.42	86.50	1.51	1.52	1179.83	96.20	11.08	0.31
100	2012	4.8	1	586	3.77	3.27	35.32	86.50	1.51	1.57	1069.6	97.60	12.79	0.13
101	2012	18.4	6	3471	1.96 ± 1.31	2.62 ± 1.35	44.57 ± 12.27	74.83 ± 1.24	0.99 ± 0.08	1.03 ± 0.08	925.70 ± 135.99	77.8 ± 6.5	8.27 ± 0.97	0.08 ± 1.05
102	2012	7.8	5	1110	1.00 ± 0.28	3.79 ± 1.11	57.25 ± 7.20	81.50 ± 1.24	1.53 ± 0.08	1.39 ± 0.03	1347.86 ± 97.84	96.6 ± 1.0	11.47 ± 0.45	0.07 ± 1.02
103	2013	67.0	20	11576	1.81 ± 0.21	2.42 ± 1.06	53.21 ± 10.36	52.60 ± 1.24	1.19 ± 0.08	1.34 ± 0.05	1029.84 ± 47.46	94.4 ± 1.2	9.90 ± 0.84	0.12 ± 1.02
104	2013	61.0	11	9131	3.20 ± 0.40	1.85 ± 1.12	34.05 ± 5.10	76.05 ± 1.24	0.86 ± 0.08	0.86 ± 0.10	850.75 ± 31.84	64.6 ± 4.0	13.17 ± 0.41	0.10 ± 1.01
105	2013	56.3	14	9433	2.40 ± 0.43	1.64 ± 1.14	32.94 ± 8.66	72.43 ± 1.24	0.75 ± 0.08	0.76 ± 0.07	782.34 ± 54.74	56.7 ± 4.9	13.06 ± 0.63	0.08 ± 1.01
106	2013	56.2	9	9789	4.33 ± 0.40	2.27 ± 1.18	36.79 ± 4.22	88.94 ± 1.24	0.91 ± 0.08	0.86 ± 0.13	1031.26 ± 60.91	65.3 ± 6.1	14.59 ± 0.52	0.17 ± 1.02

107	2013	55.3	17	7084	1.71 ± 0.20	2.50 ± 1.06	41.10 ± 9.26	81.82 ± 1.24	1.44 ± 0.08	1.40 ± 0.07	1267.93 ± 78.76	86.9 ± 3.4	15.93 ± 0.70	0.08 ± 1.01
108	2013	71.1	17	10869	2.78 ± 0.44	1.93 ± 1.16	31.81 ± 8.33	76.91 ± 1.24	0.83 ± 0.08	0.87 ± 0.08	734.37 ± 73.91	51.9 ± 5.9	10.07 ± 0.44	0.07 ± 1.01
109	2013	52.2	10	6446	3.55 ± 0.64	3.86 ± 1.04	51.32 ± 8.09	82.75 ± 1.24	1.53 ± 0.08	1.43 ± 0.02	1224.30 ± 50.56	96.7 ± 0.9	11.12 ± 0.37	0.19 ± 1.03
110	2014	63.6	12	11767	3.52 ± 0.29	1.75 ± 1.19	33.21 ± 10.13	83.79 ± 1.24	0.81 ± 0.08	0.79 ± 0.12	887.37 ± 120.00	48.9 ± 7.7	14.74 ± 0.55	0.11 ± 1.02
111	2014	70.3	11	16866	4.52 ± 0.70	1.42 ± 1.19	28.38 ± 6.17	85.14 ± 1.24	0.80 ± 0.08	0.70 ± 0.05	560.28 ± 76.53	31.3 ± 6.3	9.38 ± 0.52	0.08 ± 1.01
112	2014	64.5	12	8401	3.64 ± 0.19	3.24 ± 1.10	43.25 ± 3.52	85.21 ± 1.24	1.22 ± 0.08	1.29 ± 0.13	1093.26 ± 23.72	82.8 ± 2.9	10.94 ± 0.38	0.15 ± 1.01
113	2014	64.1	12	10201	3.31 ± 0.22	2.47 ± 1.16	43.06 ± 6.58	84.92 ± 1.24	0.91 ± 0.08	0.90 ± 0.10	1110.93 ± 62.71	67.7 ± 5.1	11.03 ± 0.55	0.12 ± 1.01
114	2014	62.5	9	8085	4.40 ± 0.29	3.03 ± 1.09	43.71 ± 5.28	87.00 ± 1.24	1.48 ± 0.08	1.41 ± 0.09	1308.86 ± 46.55	90.6 ± 2.9	13.57 ± 0.42	0.19 ± 1.01
115	2015	20.2	6	2861	2.28 ± 1.31	3.66 ± 1.21	60.56 ± 12.14	70.83 ± 1.24	1.31 ± 0.08	1.31 ± 0.05	1109.63 ± 106.10	90.0 ± 4.3	8.42 ± 0.97	0.12 ± 1.07
116	2015	24.1	15	3224	0.85 ± 0.11	3.43 ± 1.09	60.84 ± 7.99	74.67 ± 1.24	1.32 ± 0.08	1.40 ± 0.07	1384.50 ± 42.13	95.3 ± 1.0	10.14 ± 0.31	0.05 ± 1.01
117	2015	14.7	1	2929	7.91	0.95	24.34	87.00	0.41	0.42	600.33	24.60	11.10	0.14
118	2016	3.0	1	1261	2.97	1.28	37.83	85.50	0.49	0.50	878.79	30.50	9.43	0.08
119	2016	34.5	5	10593	5.16 ± 1.28	1.32 ± 2.27	28.92 ± 10.38	85.90 ± 2.24	0.51 ± 0.08	0.53 ± 0.06	537.30 ± 114.87	26.1 ± 6.8	6.05 ± 0.31	0.07 ± 2.02
120	2016	37.7	5	8599	5.65 ± 1.00	2.28 ± 2.47	42.32 ± 9.13	86.40 ± 2.24	0.71 ± 0.08	0.70 ± 0.21	974.43 ± 119.08	52.9 ± 12.2	6.68 ± 0.21	0.13 ± 2.02
121	2017	38.1	28	4243	0.60 ± 0.07	2.64 ± 2.07	49.76 ± 10.22	71.45 ± 2.24	1.31 ± 0.08	1.33 ± 0.06	1214.23 ± 62.26	89.1 ± 2.5	13.60 ± 0.39	0.03 ± 2.00
122	2017	26.9	4	3805	4.35 ± 1.05	2.63 ± 2.39	38.13 ± 8.84	85.25 ± 2.24	0.82 ± 0.08	0.88 ± 0.17	954.79 ± 115.36	68.3 ± 11.3	9.82 ± 0.83	0.11 ± 2.01
123	2017	29.8	5	4625	3.65 ± 0.18	1.73 ± 2.14	30.36 ± 4.80	84.20 ± 2.24	0.58 ± 0.08	0.61 ± 0.04	692.29 ± 83.95	40.9 ± 5.4	9.55 ± 0.77	0.08 ± 2.01
124	2017	5.8	1	910	4.03	2.80	43.88	85.50	0.87	0.83	1188.76	75.90	12.26	0.15
125	2017	25.8	8	4471	1.98 ± 0.53	2.58 ± 2.37	42.14 ± 13.01	81.63 ± 2.24	1.00 ± 0.08	1.07 ± 0.18	986.35 ± 113.43	66.9 ± 11.1	11.68 ± 0.53	0.06 ± 2.01
126	2017	26.8	4	6700	4.08 ± 1.23	1.48 ± 2.63	32.08 ± 14.78	85.88 ± 2.24	0.62 ± 0.08	0.61 ± 0.13	614.42 ± 178.66	30.1 ± 14.7	7.44 ± 0.51	0.05 ± 2.02
127	2017	3.3	1	495	2.17	2.46	39.01	87.00	0.58	0.64	884.57	55.60	11.75	0.07
128	2017	7.4	2	2315	2.79 ± 0.66	1.64 ± 2.15	47.86 ± 0.03	83.75 ± 2.24	0.46 ± 0.08	0.44 ± 0.03	1025.08 ± 26.57	36.7 ± 4.4	8.60 ± 0.24	0.11 ± 2.03
129	2017	74.6	14	14270	3.68 ± 0.62	2.28 ± 2.21	43.99 ± 8.02	83.57 ± 2.24	0.93 ± 0.08	0.92 ± 0.10	838.98 ± 100.35	50.7 ± 8.1	10.31 ± 0.71	0.12 ± 2.03
130	2018	14.8	2	474	2.98 ± 0.04	2.18 ± 2.39	12.24 ± 4.43	85.75 ± 2.24	0.79 ± 0.08	0.85 ± 0.14	235.31 ± 57.78	42.5 ± 7.3	7.46 ± 0.16	0.02 ± 2.01
131	2018	8.3	2	987	3.00 ± 0.55	4.23 ± 2.19	48.49 ± 4.13	86.50 ± 2.24	1.46 ± 0.08	1.39 ± 0.02	1111.85 ± 14.28	97.2 ± 0.1	9.20 ± 0.84	0.14 ± 2.02
132	2018	43.0	17	7715	1.69 ± 0.42	3.10 ± 2.10	57.36 ± 7.70	62.29 ± 2.24	1.18 ± 0.08	1.36 ± 0.03	938.14 ± 68.64	94.0 ± 1.3	7.53 ± 0.40	0.12 ± 2.03
133	2018	36.8	7	4995	3.80 ± 0.31	3.26 ± 2.15	42.81 ± 4.95	85.21 ± 2.24	1.59 ± 0.08	1.60 ± 0.04	1132.22 ± 64.33	91.1 ± 4.3	12.47 ± 0.27	0.17 ± 2.01
134	2019	119.6	18	29785	4.66 ± 0.34	2.10 ± 2.21	43.12 ± 5.01	83.14 ± 2.24	0.73 ± 0.08	0.74 ± 0.09	896.14 ± 52.11	50.6 ± 6.0	8.92 ± 0.21	0.14 ± 2.01
135	2019	74.2	3	2485	3.92 ± 0.55	2.63 ± 2.17	38.41 ± 5.81	86.83 ± 2.24	1.58 ± 0.08	1.64 ± 0.06	960.75 ± 54.44	72.0 ± 3.0	12.73 ± 0.90	0.14 ± 2.02
136	2019	81.3	11	14798	5.19 ± 0.52	1.56 ± 2.12	28.63 ± 6.46	87.68 ± 2.24	1.93 ± 0.08	1.81 ± 0.05	461.64 ± 54.68	27.3 ± 3.8	12.55 ± 0.31	0.10 ± 2.02
137	2019	61.3	2	1673	3.14 ± 0.20	2.16 ± 2.33	39.30 ± 2.28	82.75 ± 2.24	1.77 ± 0.08	1.65 ± 0.13	1073.07 ± 37.92	62.9 ± 10.6	17.18 ± 1.11	0.13 ± 2.00
138	2019	101.5	15	12994	4.32 ± 0.41	3.24 ± 2.08	45.51 ± 6.60	93.03 ± 2.24	1.83 ± 0.08	1.91 ± 0.01	994.12 ± 67.08	93.5 ± 3.1	13.94 ± 0.21	0.17 ± 2.01
**Mean**		32.2 ± 2.6	7.0 ± 0.6	4338 ± 394	2.72 ± 0.07	2.80 ± 0.03	43.77 ± 0.39	78.52 ± 0.50	1.20 ± 0.01	1.22 ± 0.01	979.27 ± 10.62	78.2 ± 0.74	10.24 ± 0.11	0.10 ± 0.00
**Total**		4476.8	970	602938										

Supplementary table S3. Annual dive metrics for Australian fur seals (*Arctocephalus pusillus doriferus*) from Kanowna Island.

Year	Deployment duration (d)	No. of trips analysed	No. of dives analysed	Trip duration (d)	Dive duration (min)	Proportion of time spent diving (%)	Max depth (m)	Descent rate (m s-1)	Ascent rate (m s-1)	Dive rate (m h-1)	Proportion of benthic diving (%)	Foraging Trip Success Index	Foraging Trip Efficiency Index
1998	36.31 ± 19.59	28	26283	4.92 ± 0.48	3.14 ± 0.11	41.97 ± 1.45	83.29 ± 0.65	1.34 ± 0.06	1.42 ± 0.07	987.87 ± 46.15	85.1 ± 3.8	12.06 ± 0.81	0.21 ± 0.02
1999	85.96 ± 13.39	47	32608	3.28 ± 0.32	2.65 ± 0.08	40.76 ± 2.04	73.86 ± 0.62	1.19 ± 0.03	1.23 ± 0.05	812.73 ± 43.77	80.4 ± 3.0	8.96 ± 0.35	0.11 ± 96.02
2000	-	-	-	-	-	-	-	-	-	-	-	-	-
2001	13.71	3	1862	3.76 ± 0.69	2.62 ± 0.22	29.06 ± 2.16	119.33 ± 0.33	1.39 ± 0.10	1.54 ± 0.15	629.55 ± 90.72	57.2 ± 8.6	9.01 ± 1.52	0.08 ± 01.02
2002	39.12 ± 16.55	26	17353	3.95 ± 0.46	3.59 ± 0.11	42.08 ± 1.82	83.08 ± 0.74	1.30 ± 0.04	1.27 ± 0.04	910.47 ± 38.93	84.6 ± 2.0	8.23 ± 0.39	0.15 ± 23.02
2003	36.27 ± 12.80	96	42980	1.71 ± 0.16	2.42 ± 0.09	45.31 ± 1.42	64.89 ± 0.80	1.22 ± 0.03	1.27 ± 0.04	909.07 ± 36.59	78.6 ± 2.5	10.41 ± 0.31	0.07 ± 4.01
2004	8.73	1	634	4.76	3.34	30.95	84.00	1.43	1.49	833.89	93.1	10.55	0.13
2005	52.21 ± 13.65	62	26118	2.33 ± 0.26	2.94 ± 0.09	39.38 ± 1.42	87.35 ± 0.22	1.15 ± 0.03	1.15 ± 0.04	827.05 ± 31.16	81.4 ± 2.3	7.95 ± 0.43	0.06 ± 95.01
2006	20.25 ± 9.52	18	13001	4.02 ± 0.48	2.80 ± 0.08	36.79 ± 1.95	83.83 ± 0.40	1.44 ± 0.09	1.43 ± 0.09	1096.37 ± 69.16	87.3 ± 3.7	14.30 ± 0.98	0.15 ± 3.02
2007	35.72 ± 10.67	71	30520	2.13 ± 0.19	3.08 ± 0.06	45.10 ± 1.43	82.01 ± 0.33	1.12 ± 0.03	1.15 ± 0.04	1027.39 ± 45.32	78.0 ± 2.3	8.35 ± 0.30	0.06 ± 35.01
2008	16.20 ± 4.39	43	15901	2.11 ± 0.27	3.53 ± 0.06	50.30 ± 1.77	80.43 ± 0.46	1.40 ± 0.03	1.41 ± 0.01	1129.62 ± 37.19	89.8 ± 0.7	9.74 ± 0.48	0.08 ± 74.01
2009	21.39 ± 7.50	74	45637	2.92 ± 0.29	2.83 ± 0.07	44.20 ± 1.14	78.54 ± 0.66	1.20 ± 0.03	1.20 ± 0.04	1037.96 ± 31.11	82.7 ± 1.5	10.42 ± 0.42	0.11 ± 4.01
2010	10.58 ± 2.57	9	5411	3.02 ± 0.53	3.20 ± 0.30	44.33 ± 2.06	75.39 ± 0.62	1.28 ± 0.06	1.27 ± 0.10	876.37 ± 65.36	82.8 ± 5.3	8.40 ± 0.67	0.11 ± 40.02
2011	21.98 ± 4.95	74	26187	1.72 ± 0.19	3.16 ± 0.08	49.72 ± 1.58	79.13 ± 0.07	1.30 ± 0.03	1.28 ± 0.03	1120.02 ± 46.72	84.5 ± 2.4	9.26 ± 0.45	0.07 ± 26.01
2012	15.74 ± 2.58	69	41168	2.59 ± 0.27	3.12 ± 0.10	45.92 ± 1.23	78.45 ± 0.07	1.23 ± 0.04	1.24 ± 0.04	1058.42 ± 32.33	84.3 ± 2.3	10.18 ± 0.28	0.11 ± 1.01
2013	59.86 ± 2.60	98	64328	2.61 ± 0.16	2.31 ± 0.07	40.65 ± 1.19	73.77 ± 0.56	1.08 ± 0.04	1.10 ± 0.04	984.40 ± 30.80	74.6 ± 2.3	12.35 ± 0.34	0.11 ± 3.01
2014	65.00 ± 1.37	56	55320	3.84 ± 0.18	2.36 ± 0.12	38.21 ± 1.22	85.12 ± 0.22	1.03 ± 0.06	1.00 ± 0.06	982.89 ± 46.53	63.5 ± 3.7	11.89 ± 0.34	0.13 ± 8.01
2015	19.67 ± 2.73	22	9014	1.56 ± 0.48	3.38 ± 0.14	59.11 ± 2.50	74.18 ± 0.55	1.28 ± 0.06	1.33 ± 0.07	1273.90 ± 57.16	90.6 ± 3.4	9.71 ± 0.37	0.08 ± 71.02
2016	25.07 ± 11.09	11	20453	5.18 ± 0.73	1.75 ± 0.28	35.82 ± 3.33	86.09 ± 0.36	0.60 ± 0.08	0.60 ± 0.10	767.04 ± 97.19	38.7 ± 7.2	6.64 ± 0.34	0.10 ± 64.01
2017	26.50 ± 7.26	67	41834	2.21 ± 0.25	2.39 ± 0.09	44.14 ± 1.38	78.64 ± 0.07	1.03 ± 0.05	1.04 ± 0.05	1007.41 ± 44.97	67.8 ± 3.6	11.59 ± 0.34	0.07 ± 5.01
2018	22.17 ± 7.40	30	15151	2.44 ± 0.30	3.21 ± 0.11	49.61 ± 2.47	72.53 ± 0.77	1.28 ± 0.04	1.37 ± 0.04	952.97 ± 56.55	89.9 ± 2.7	8.76 ± 0.46	0.13 ± 76.02
2019	85.54 ± 8.66	65	71175	4.28 ± 0.22	2.55 ± 0.11	39.22 ± 1.08	85.28 ± 0.80	1.41 ± 0.06	1.43 ± 0.06	819.40 ± 36.72	64.4 ± 3.7	11.13 ± 0.35	0.13 ± 1.01

Supplementary table S4. Annual mean local-scale environmental variables and large-scale climate variables used in GAMM analyses.

Year	Winter Chl-a	Spring Chl-a	Winter wind-u component	Spring wind-u component	Winter SSTa	Spring SSTa	Winter SSHa	IOD	SOI	SAM
1996	-	-	-	5.08	-	2.05	-	-0.21	5.69	-0.41
1997	-	0.53	-	3.39	-	2.00	-	0.67	-11.67	0.35
1998	0.57	0.57	3.13	3.18	2.61	1.91	0.04	0.09	-1.08	1.60
1999	0.53	0.46	3.16	1.96	2.14	1.13	0.08	0.19	7.95	1.27
2000	0.55	0.48	4.12	3.14	2.00	1.59	0.07	0.23	7.80	0.26
2001	0.60	0.49	2.70	3.10	1.65	1.29	0.05	0.13	0.48	0.54
2002	0.51	0.45	5.82	4.59	1.60	1.79	0.05	0.17	-6.15	-0.51
2003	0.61	0.55	4.83	4.06	1.85	2.12	0.07	0.21	-3.18	0.08
2004	0.53	0.44	5.47	3.64	2.15	1.55	0.06	0.11	-4.78	0.35
2005	0.48	0.51	4.46	2.75	1.52	1.13	0.05	-0.04	-3.57	0.28
2006	0.53	0.46	3.41	3.80	2.21	1.56	0.02	0.33	-2.11	0.50
2007	0.57	0.49	3.40	2.95	1.71	1.28	0.02	0.35	1.29	-0.27
2008	0.51	0.50	3.76	3.57	1.68	1.63	0.03	0.31	10.12	0.95
2009	0.54	0.42	4.05	2.68	1.81	1.42	0.03	0.29	-0.33	0.12
2010	0.54	0.55	3.93	3.11	1.73	2.14	0.04	0.21	9.82	1.15
2011	0.85	0.57	3.50	2.08	2.29	1.61	0.05	0.44	13.30	0.24
2012	0.69	0.51	4.07	3.88	2.06	1.98	0.04	0.36	-0.77	0.82
2013	0.69	0.59	3.81	4.29	1.27	1.26	0.07	0.12	3.98	0.21
2014	0.58	0.38	3.95	3.49	1.14	1.36	0.09	0.15	-3.03	0.51
2015	0.65	0.45	4.31	2.35	2.71	1.88	0.03	0.41	-11.23	1.61
2016	0.70	0.58	4.96	4.25	2.07	2.08	0.07	0.01	-3.08	0.76
2017	0.63	0.49	5.09	3.30	1.75	1.63	0.05	0.51	2.15	0.55
2018	0.74	0.45	5.21	2.10	2.02	1.84	0.09	0.43	0.95	0.76
2019	0.76	0.77	5.85	4.06	1.92	2.38	0.07	0.59	-7.11	0.21
Mean ± SE	0.61 ± 0.02	0.51 ± 0.02	4.23 ± 0.19	3.37 ± 0.17	1.90 ± 0.08	1.69 ± 0.07	0.05 ± 0.00	0.25 ± 0.04	0.23 ± 1.33	0.50 ± 0.11
